# The Spread of Inequality

**DOI:** 10.1371/journal.pone.0024683

**Published:** 2011-09-21

**Authors:** Deborah S. Rogers, Omkar Deshpande, Marcus W. Feldman

**Affiliations:** 1 Department of Biology, Stanford University, Stanford, California, United States of America; 2 International Human Dimensions Programme, United Nations University, Bonn, Germany; 3 Department of Computer Science, Stanford University, Stanford, California, United States of America; University of Maribor, Slovenia

## Abstract

The causes of socioeconomic inequality have been debated since the time of Plato. Many reasons for the development of stratification have been proposed, from the need for hierarchical control over large-scale irrigation systems to the accumulation of small differences in wealth over time via inheritance processes. However, none of these explains how unequal societies came to completely displace egalitarian cultural norms over time. Our study models demographic consequences associated with the unequal distribution of resources in stratified societies. Agent-based simulation results show that in constant environments, unequal access to resources can be demographically destabilizing, resulting in the outward migration and spread of such societies even when population size is relatively small. In variable environments, stratified societies spread more and are also better able to survive resource shortages by sequestering mortality in the lower classes. The predictions of our simulation are provided modest support by a range of existing empirical studies. In short, the fact that stratified societies today vastly outnumber egalitarian societies may not be due to the transformation of egalitarian norms and structures, but may instead reflect the more rapid migration of stratified societies and consequent conquest or displacement of egalitarian societies over time.

## Introduction

Inequalities in socioeconomic status are increasing sharply within and between societies [1]–[3]. But human societies have not always been stratified. In this study we propose and test a demographic mechanism that may explain how stratified societies spread and became the dominant form of societal organization.

It has been suggested that early foraging societies rigorously enforced social and economic “leveling mechanisms” to prevent any individual or group from acquiring more status, authority, or resources than others [4]–[10]. In fact, one of the central adaptations during the course of human evolution may have been the suppression of older dominance instincts through the enforcement of these egalitarian cultural norms [6]. Whether or not this was the case, it is generally accepted that early societies were less complex and more equal than societies arising over the past 10,000 years [7].

How did stratified societies—those with institutionalized inequality—spread and become the dominant form of societal organization during the Neolithic period? Mid-twentieth-century explanations assumed that population growth led to the need for agriculture, which generated surplus and required managers and other specialized roles, leading naturally to various classes [11]–[13]. When resource depletion necessitated further expansion, conflict and conquest resulted in even greater hierarchy. More recent explanations of socioeconomic stratification are generally individualistic (natural tendencies toward selfishness and dominance impart individual selective advantage) [14]–[16], group adaptive (socioeconomic stratification promotes cooperation or confers economic and organizational efficiencies that enable societies to cope with new technologies, larger populations, competition and conflict) [17]–[23], or mechanistic (patchy landscapes, private property and inheritance mechanisms lead to the accumulation of small differences in wealth over time) [24]–[26].

While each of these explanations contributes insights on why the shift towards stratification may have taken place, or on mechanisms by which inequality was self-perpetuating, none develops the dynamics of the process by which unequal societies displaced more egalitarian societies. Why did early pastoralists and agriculturalists relinquish a communal approach to property and cease to enforce the leveling mechanisms that had previously prevented individual differences and stochastic economic shocks from resulting in permanent inequalities? Empirical data on cultural transmission indicate that norms, values, social structures and other foundational features of culture tend to be transmitted conservatively and vertically within groups [27]–[31]. Such communally-held traits are not likely to be altered by individual choices or by transmission from group to group. However, they may instead spread through demic diffusion; *i.e.* population growth or migration of the groups carrying the culture. Demographic studies have linked fertility and mortality with access to food resources [32], [33]. Because egalitarian and stratified societies allocate resources—including food—differently, the two cultural strategies could have strong implications for the demography and thus the expansion of populations bearing the two cultural types.

We hypothesize, therefore, that the spread of socioeconomic stratification may have been a result of cultural change via demic diffusion. In other words, socioeconomic stratification may have spread across the globe over the past several thousand years, not because it provided apparent advantages that led to its adoption by egalitarian cultures, but simply because it altered demographic outcomes in ways that produced an increase in frequency of stratified populations, through population expansion or the outward migration of populations in search of additional territory and resources.

To explore this hypothesis, we developed an explicit quantitative demographic model of the process, ran various agent-based simulation trials, developed predictions based on model results, and tested some of these using available data. As with population genetic models of the spread of random mutations, our model is not intended to show how inequality developed between individuals, but to explore a basic demographic mechanism that may have caused the spread of such societies once stratification emerged. Our ultimate objective is to determine whether demic diffusion could have produced the global shift from egalitarian to stratified societies over time, and, where possible, to test the predictions of this model against real world data.

## Results

**Figure 1:**
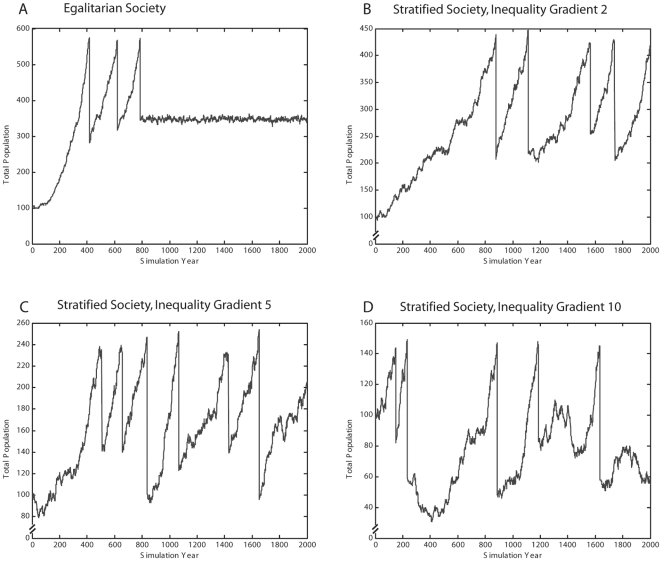
Typical egalitarian and stratified populations in constant environment with baseline parameter values. Egalitarian populations (A) are able to stabilize, although no logistic or density-dependent growth function was used, while stratified populations (B, C, D) cannot stabilize because upper classes continue to thrive even as resources are being depleted.

Our simulation trials showed that stratified populations in constant environments exhibited more demographic instability, crises and extinctions than did egalitarian populations. Figure 1 shows typical population trajectories for egalitarian and stratified societies over 2000 years. Egalitarian populations are eventually able to stabilize, not because of density-dependent growth but because fertility, mortality, and resource productivity achieve a balance. This is an unexpected outcome in a complex system. Reaching this balance appears to depend on the stochastically determined magnitude of the rebound following a population crash.

Stratified populations were never able to stabilize because the upper classes continued to thrive even as resources were being depleted and the population was headed for trouble. In other words, stratification disrupted stabilizing feedback in the system. However, stratified populations did not cause more resource depletion, as we had originally predicted on the basis of excess resource consumption by upper classes. Instead, stratified populations in our simulation depleted the resource base significantly less because high mortality rates in lower classes kept the total population relatively low compared to carrying capacity.

Analysis of variance and multiple comparisons tests based on 100 trials for each type of society show that the amount of time spent in a state of population stability was significantly greater for egalitarian societies. Stability was defined as a time period of 100 or more years during which population size varied by less than ±5% on either side of the mean. Multiple comparisons tests showed that there were significantly fewer demographic crises or “population crashes” (an event in which the population loses at least 25% of its size in one year or successive years of population decline) for egalitarian groups than for stratified groups. There were also significantly fewer extinctions for egalitarian groups, based on the non-parametric rank order Kolmogorov-Smirnov Test. Each trial run had a different outcome due to stochasticity of the demographic and ecological processes in the simulation.

After establishing these baseline parameters and results, we conducted an analysis of the sensitivity of the results to the parameter values we used. The parameter values and results for each sensitivity test are shown in Supporting Information S1. Within a reasonable range of parameter values, results were robust. Egalitarian populations were always more stable at every parameter value except in trials when current resource amount determines half or more of the next year's productivity (see Supporting Information S1). This appears to lead to a very unstable environment: once depletion begins, it exerts a positive feedback effect and causes a rapid crash of egalitarian populations.

Egalitarian populations always have fewer population crashes except in trials with a base female fertility rate of 0.135 (see Supporting Information S1). This anomalous spot in parameter space seems to be just high enough to cause population growth and thus resource depletion, but too low to allow sufficient recovery.

Egalitarian populations are almost always much less likely to go extinct, except in trials when resource amount determines half or more of the next year's productivity (discussed above), and in trials with a base annual female fertility rate of >0.18 (see Supporting Information S1), which apparently causes egalitarian populations to grow too large, thus depleting resources and resulting in rapid mortality.

**Figure 2:**
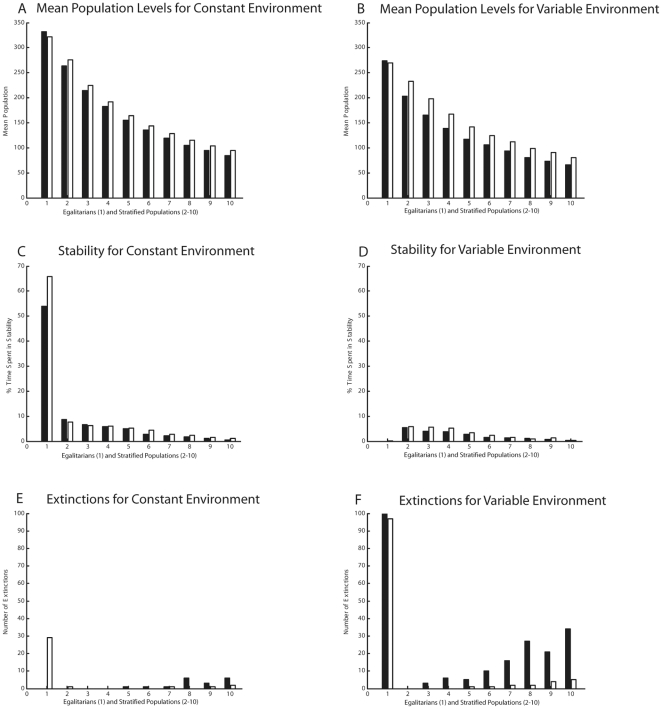
Egalitarian and stratified populations in constant and variable environments, with and without storage of surplus. Black bars show results without storage and white bars with storage. Egalitarians have larger, more stable populations in constant environments (A, C), while storage causes them to overshoot carrying capacity by a greater extent and go extinct (E). In variable environments, egalitarian populations are unstable and go extinct (B, D, F). Stratified groups have smaller, less stable populations in constant environments (A, C), and are more likely to go extinct (E). In variable environments, stratified populations are protected against extinctions because mortality is sequestered in the lower classes; storage of surplus gives further protection (B, D, F).

We next altered the simulation to investigate how variable environments (in which productivity varies from year to year) and the storage of surplus resources (to be used during times of shortage) would affect outcomes (see Figure 2). In general, stratified groups have a lower mean population size than egalitarians, both because the upper classes reduce the carrying capacity by taking extra resources, and because lower classes suffer high levels of mortality. Resource storage appears to raise the population size slightly for stratified groups but not for egalitarians. In constant environments, egalitarians have no extinctions without storage, but storage causes them to overshoot carrying capacity by a greater amount, leading to extinctions. In variable environments, egalitarians have very unstable populations and go extinct rapidly; storage does not afford them protection. Stratification appears to protect populations against environmental variation because mortality in bad years is sequestered in the lower classes, allowing upper classes to survive, while in egalitarian societies the entire population is at risk. Storage confers some additional protection to stratified societies in variable environments.

**Figure 3:**
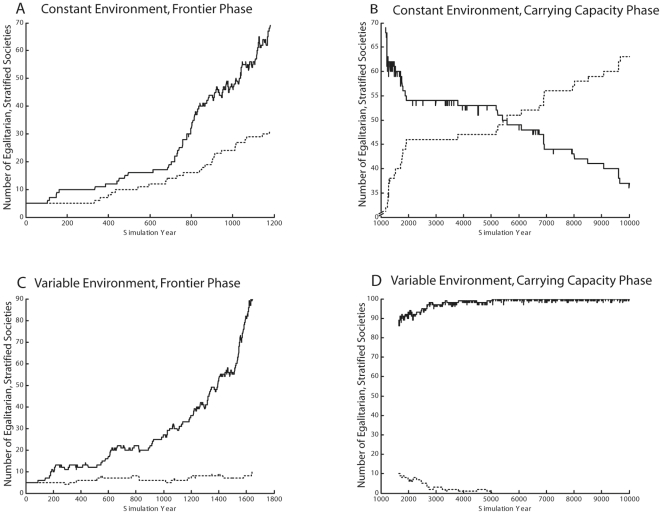
Representative migration competition outcomes in constant and variable environments. Solid lines show number of sites held by stratified societies, and dotted lines show number of sites held by egalitarian societies. The two plots on the left (A, C) show rate of occupying sites during the “frontier phase” when uninhabited sites are still available, while the two plots on the right (B, D) show what happens after all sites are occupied (“carrying capacity phase”), and thus expansion can only take place when a site opens up because another population goes extinct. These plots show results using the population decline trigger optimal values (loss of a threshold fraction of population), but results for the other two migration triggers (resource deprivation threshold for individuals, and resource depletion threshold for sites) are similar. Stratified societies always migrate outward more frequently, and thus take over quickly (A, C). Over the long term, in comparison with egalitarian societies they experience a higher rate of group extinctions in constant environments (B), and a lower rate in variable environments (D).

Finally, we ran a series of migration trials to see which society type—egalitarian or stratified—filled previously unoccupied sites most rapidly. For each migration event, 65 individuals (or half the population, if fewer than 130) moved to an unoccupied site; no conflict occurred. Migration events were triggered when a group exceeded some threshold value for group mortality, for individual resource deprivation, or for resource depletion. Figure 3 shows that although stratified groups have smaller mean populations and are more likely to go extinct in constant environments, they migrate more often and thus fill unoccupied sites more rapidly—for all three triggers—in both constant and variable environments, as long as open sites are still available (“frontier phase”). The simulation did not allow migration into or conflict with already-occupied sites, although sites at which an existing population had gone extinct could be reoccupied. Thus, once all sites were occupied (“carrying capacity phase”), the original trial dynamics of stability and extinctions determined how many sites were held or lost by egalitarian and stratified populations. In the real world, of course, conflict and conquest allowed stratified groups to continue their expansion even after all sites were occupied.

## Discussion

Our simulation results support the hypothesis that socioeconomic stratification may have spread due to its effects on the demography of small groups—i.e. by demic diffusion—rather than cultural adoption. While demic diffusion has already been indicated as a mechanism for the displacement of hunter-gatherers by more rapidly-growing agricultural populations [34]–[36], we do not specify differences in mode of subsistence, and the demic diffusion shown in our model is based not on population expansion but rather on migration due to population instability. If demic diffusion were simply a function of population growth, then in constant environments egalitarians would take over sites available for colonization much more rapidly. In our demographic simulation, however, three different plausible migration triggers all lead stratified groups to take over unoccupied sites faster.

In constant environments, inequalities in resource allocation appear to disrupt the feedback between population growth and resource depletion, preventing stratified groups from achieving an equilibrium size and driving them to migrate more despite their smaller populations. In variable environments, stratified groups migrate more and are less likely to go extinct than egalitarian groups. While rapid migration and protection against extinction in variable environments may be viewed as an adaptive advantage of stratification, it is more probably a result of individual selection, as certain individuals survive at the expense of resource deprivation and mortality for others.

A recent paper linking political egalitarianism with the extreme variability of the Last Glacial environment argues that scarce and unpredictable resources enforced behavioral constraints such as small band size, mobility, and nonacquisitiveness, thus blocking establishment of wealth hierarchies until the climate stabilized during the Holocene [4]. Our simulation results do not contradict this proposal—because we do not include these constraints—but may indicate that once the severe constraints of the Last Glacial were relaxed during the Holocene, the demographic consequences of stratification were able to contribute to its spread. In a more moderate environment, cultural and behavioral responses to resource shortage can include increased labor or productivity, resource intensification, shortened reproductive age span, fertility reduction, infanticide and others. Once all habitable sites are occupied in a region, conflict can come into play as resource-deprived groups attempt to acquire more territory. Migration-driven conflict is likely to have been an important behavioral response by which stratified societies came to dominate the landscape during the Holocene.

Tuljapurkar and others have developed a complex demographic model that incorporates feedbacks between food supply, human fertility and mortality rates, and labor availability in early Polynesian agricultural populations [37]–[39]. They found that fertility control decreases population growth but increases individual well-being, while increased labor productivity increases the equilibrium population size but does not improve individual well-being. The presence of an upper class to which tribute was paid had the effect of reducing the equilibrium population size, much as stratification resulted in smaller populations in our simulation.

Structure or stratification of populations have been identified as important keys to game theoretic models on the evolution of cooperation [23]. Perc and Szolnoki investigated the impact of social diversity (inequality based on a uniform, exponential, or power law distribution of benefits) on a spatial network-based Prisoner's Dilemma game [22]. Counterintuitively, inequality (the presence of high-ranking cooperative individuals) promoted the evolution of cooperation by providing successful nodes around which other cooperators could cluster. Similarly, Santos et al. found that for graph-based evolutionary Public Goods Games, heterogeneity in the number, size and cost of games in which individuals participated promoted the evolution of cooperation by providing a strong advantage to cooperators at the hubs of relatively large groups of interacting individuals [17]. Assuming that cooperation is advantageous for groups, this suggests another potential evolutionary mechanism for the development of stratified societies.

### Potential Objections

One potential objection to our results is that stratified populations in Neolithic and later times were much larger than those of egalitarian foragers from pre-Neolithic times. However, this can be accounted for by the increased carrying capacity brought about by the change from foraging to farming. Our simulation was designed to address differences in outcomes only for egalitarian and stratified societies with the same mode of subsistence and inherent carrying capacities.

It could also be objected that poor families are generally larger than well-off families. While this is true in societies that have undergone the modern demographic transition, the modern cultural and economic circumstances thought to drive this outcome do not pertain to older, pre-demographic-shift societies such as those investigated in our study.

Another potential objection might be that lower classes are more likely to suffer deprivation and thus to migrate, leading to a new society in which all members are derived from the same class. Petersen's classic treatise on the various types of human migration indicates that very few involve the relocation of families from only one social class (c.f. p. 260) [40]. Other than the relatively rare case of intentional emigration to establish a new type of society (e.g. Kibbutzim), to our knowledge there are no data suggesting that migrating groups alter their socioeconomic structure in the process of migration.

The parameter values we used resulted in a population growth rate (r) of approximately 0.01, before a resource limiting state is reached. While some literature suggests that early human populations could have had higher growth rates, approaching r = 0.03 [41], we ran trials with lower rates because hunter-gatherer societies are known to have limited their reproduction, and because our simulated populations went extinct with the higher fertility rates. Sellen 2001 found that fertility goes up with increasing dependence on agricultural subsistence [42]. Kirch and Rallu 2007 estimated long-term average population growth for Polynesian populations (horticulture and fishing) at <1% [43]. Gurven and Kaplan 2007 estimated population growth rates for hunter-gathers at 0–1%, for horticulturalists at >2%, and for pastoralists at >2% [44].

### Alternative Models of Inequality

There are various ways in which socioeconomic inequality can be structured and maintained. Our results were qualitatively the same for two classes as for five. When we initialized each of the five classes with 20% of the population but did not maintain the class structure over time, lower classes died off one by one, eventually leaving only one class, which functioned as an egalitarian society and rapidly stabilized its population. Our maintenance of class structure each year—pulling population in from other classes as needed—may be considered analogous to inheritance by primogeniture, in which first sons maintain the family fortune while later sons slip into lower classes.

In order to determine whether our findings were robust to a completely different model of inequality with no elements of class structure, we modified our simulation to allocate resources to individuals according to a Pareto Distribution [45]. We ran baseline trials (constant environment, no storage of surplus, no migration) using three different Pareto indices, chosen to generate distributions covering the same range of Gini inequality coefficients as our gradient analysis of class inequality. The results indicate that Pareto-unequal populations have properties that are nearly the same as those of the class societies used in our original simulation: low, unstable population size, unresponsiveness to carrying capacity, with more crashes and extinctions than those of egalitarians (see Supporting Information S1). The one exception occurred in populations with the most extreme Pareto index: although highly unstable, these populations were so small that they rarely exceeded carrying capacity or crashed. In short, inequality in access to resources, whether based on class structure or Pareto Distribution to individuals, results in similar demographic instability.

To better understand the sources of demographic instability generated by our simulation, we developed a simple recursive equation for logistic population growth, with a variance in carrying capacity, *k*. The results of iterating this equation are shown in Supporting Information S1. As variance of the carrying capacity increased, the trajectory of the population over time became increasingly irregular, and eventually began to generate population extinctions, just as we saw with increasing the resource allocation multiplier for our simulated stratified societies.

Variance in *k* is intended as a substitute for the effects of increased stratification (that is, increased variation in access to resources by members of the population). While this sheds some light on the increasingly unstable populations we see with stratification, it is an oversimplification. There are at least four sources of variance affecting resource availability in our full simulation: natural variance in environmental productivity from year to year (for variable environment trials), variance in resource availability between years when the population is below carrying capacity and years when the resource base becomes depleted, variance between classes in the stratified societies, and variance between egalitarians and the nine levels of stratified societies (gradient multipliers 2 through 10). These variances interact to give the unpredictable outcomes we see, especially those which drive outward migration. Although some aspects of the population instability in our simulation are captured by this equation, the interesting story actually lies in the mathematically intractable complexity.

### Testing Predictions

These simulation trials are not intended to serve as a test of our proposed hypothesis. Instead, the simulation is designed to clarify and refine our hypotheses, ensuring internal consistency. The ultimate test will come from comparing predictions generated by the simulation to real-world data and to the ethnographic and historical record.

If our model were valid, we would expect to see higher migration rates from countries with greater inequality, all other factors being equal. And indeed there is some evidence for this. After controlling for a country's wealth (GNP per capita) and unemployment rate, Liebig and Sousa-Poza (2004) found a strong and significant relationship between income inequality (Gini coefficient and other income disparity measures) and propensity to emigrate out of 23 countries (survey of 28,000 individuals) [46]. Stark (2006) developed a model to explain these results, showing that if total income is held constant, total relative deprivation, and thus, according to his model, desire to emigrate, is positively related to income inequality (Gini coefficient) [47].

Links between inequality and conflict have been sought for modern societies, with mixed results [48]–[53]. In an ethnographic study designed to understand determinants of conflict and peace in small-scale prehistoric societies, a clear association was found between level of stratification for 35 societies in Polynesia and level of conflict within and between these societies [54]. Although conflict was not used in our simulation, we have demonstrated an underlying demographic force that can drive territorial expansion and thus could promote conflict.

In our simulation, upper classes co-opt a disproportionate share of resources, leaving lower classes to bear the brunt of any mortality caused by resource shortages. Empirical studies suggest that this may actually occur. Historical data on changes in mortality as prices and production of food fluctuated from year to year across Europe and in China showed that in response to a 4–6% average consumption decline, the increase in mortality ranged from less than 0% (i.e. mortality actually decreased) to over 15%, depending on gender, age group, and socioeconomic class [32]. Several studies have documented health disparities in prehistoric skeletal remains [55]. Osteological data from a number of early foraging and agricultural societies show that within-group height and nutrition disparities developed along with socioeconomic inequalities [26]. A positive association between lower socioeconomic status and higher mortality has been well-documented in contemporary populations [56]–[65].

Case histories from the archeological literature on the spread of stratified societies constitute a rich source of relevant observations. As a test of the explanatory powers of our model, we summarized 15 such studies without regard to their implications for our predictions [15], [43], [54], [66]–[79]. We noted whether the following indicators, predicted by our model, were observed in conjunction with the rise of stratified societies: unequal allocation of resources, population instability, migration, conflict, storage of surplus, variable environments, and increased frequency of stratified societies through their spread rather than through internal development.

The results of this analysis (see Supporting Information S1) provide modest support for our model. Only two studies reported contradictory findings (the Levant and the Intermontane Plateau). Because both our analysis and the case studies themselves are based on qualitative assessments, there is no legitimate way to assign statistical probabilities to our findings.

In short, our hypothesis fits available data relatively well, and should be tested with additional empirical data, both contemporary and ethnographic. This is not simply an academic exercise. Socioeconomic inequality may promote conflict within and between ethnic groups, classes and societies, and drive international immigration, as mentioned above. It appears to raise prevalence of poor health, mental illness, crime, violence, and other societal ills [56], [80], [81]. Inequality reduces cultural diversity through disempowerment of local minority communities [82]. It may harm working relationships within businesses [83], inhibit economic growth in developing countries [84], reduce sustainability [85]–[88], promote corruption [89], and play a role in destabilizing economies [90]. Perhaps most dangerously, inequality erodes trust and blocks cooperative solutions to urgent social, economic and political problems [87], [91]–[93]. Understanding the causes and consequences of inequality is clearly one of the central challenges of the social sciences. Our further research on this critical topic will attempt to identify behavioral traits that tend to increase in frequency in unequal societies, as well as leverage points for shifting societies towards greater stability and social sustainability.

## Methods

We constructed an agent-based demographic simulation in which isolated human populations depend on the resources produced at each occupied site (see Supporting Information S1 for details). Each of 100 sites had just one type of society: either egalitarian or stratified. We assumed a Malthusian, pre-demographic-transition scenario where population growth eventually resulted in resource depletion, with no behavioral or cultural limitations on births. In order to understand the underlying dynamics of the population-resource interaction, we did not use a logistic or density-dependent growth function, but allowed populations to overshoot carrying capacity. We tracked all individuals by gender, age, and class. The simulation was stochastic, except for the resource productivity function, which was deterministic. All sites were assumed to be the same size and equidistant from one another; this spatial element played no overt role other than the separation of sites. Each time step in the simulation corresponded to one year.

Inequality was not defined individually in our baseline simulation. Each population was designated as egalitarian (no classes) or stratified (population divided into 5 classes, each maintained at 1/5 of the population by being redistributed yearly). Resources were then allocated to individuals based on class structure and resource availability. The factor by which resource allocation to the uppermost class exceeded that to the lowest class ranged from 2 to 10, approximating Gini inequality coefficients ranging from 0.14 to 0.42.

Because there are many ways in which a class society may be structured, we also ran the simulation using two very different models: one in which there were only two classes (again with allocation to the upper class exceeding that of the lower class by a factor ranging from 2 to 10), and one in which each individual received a different allocation, according to a Pareto Distribution (with the same range of Gini inequality coefficients, from 0.14 to 0.42). We assume that any intermediate resource allocation scheme would give results falling somewhere between the results of these three models.

A total of 40 resource units per year met the needs of one individual. As resources became limited, upper classes took their allocation before lower classes. In egalitarian societies, if more resources were available at that site, they were left untouched, while if fewer resources were available, everyone shared equally in the deprivation. No human labor productivity was included in the simulation, and mode of subsistence (foraging, pastoral, or agricultural) was not specified.

We assumed a default productivity rate (R) of 20,000 additional resource units produced per site per year. However, if the resources at the site had been depleted by the population, R was lower, as follows:
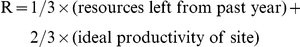
where R was capped at 20,000. This resource renewal increment was added to the previous year's resources to determine the total resources available in the present year. Each site was initialized at triple its annual productivity, i.e. at 60,000 resource units, and total resources were not allowed to exceed this amount.

Mortality and fertility rates were functions of age, class, and allocated resources, designed to approximate observed empirical relationships between survival and food availability. The parameter values we used resulted in an annual population growth rate (r), before resources became limiting, of approximately 0.01. For females in the age group 18–36, the probability of giving birth to a child in any given year was the product of a fitness metric and a resource reproduction factor, calculated using a maximum fertility rate and an elasticity function linking fertility to actual resource allocation, as follows:
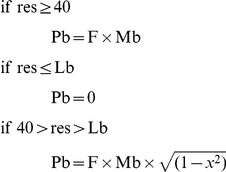
where res = resources allocated to that individual in that year

Pb = probability of that individual giving birth in that year

F = class fitness metric

Highest class: 1.005

Second class: 1.003

Third class: 1.000

Fourth class: 0.997

Lowest class: 0.995

Mb = maximum probability of giving birth in any given year

Lb = low end resource allocation at which births are no longer possible
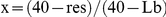



Births alternated between male and female.

From age 0–65, the probability of survival was the product of the fitness metric mentioned above, and a resource survival factor (calculated using a maximum survival rate and an elasticity function linking survival to actual resource allocation). From age 65 to 72, the probability of survival was the product of the fitness metric, the resource survival factor, and an aging factor (which diminished linearly from 1 to 0 from age 65 to age 72), as follows:
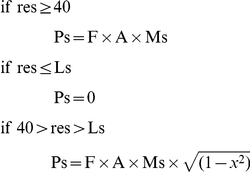
where res = resources allocated to that individual in that year

Ps = probability of that individual surviving that year

F = class fitness metric (as above)

A = age survival factor (declines linearly from 1 to 0 between age 65 to age 72)

Ms = maximum probability of surviving in any given year

Ls = low end resource allocation at which survival is no longer possible
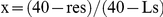



No individual survived above the age of 72.

A set of baseline parameters and initial conditions was developed that gave results encompassing a realistic range of outcomes—from general population stability through periodic extinctions. Any parameter values or initial conditions that caused most simulated populations to go rapidly extinct were rejected as not useful for understanding the system dynamics. Using these baseline parameters and initial conditions, each simulation was run for 100 populations for each of the 10 inequality gradient levels (from egalitarian through a 10-fold gradient). These 2000-year trials assumed a constant baseline productivity rate from year to year, and did not allow storage of surplus resources or migration to new sites.

Data for each of the ten inequality gradient levels were analyzed to see if there were significant differences in percent time spent in a state of population stability, number of demographic crises, probability of extinction, responsiveness to carrying capacity, and extent of resource depletion (see Supporting Information S1 for definitions and statistical tests). We conducted a visual inspection of our data to assess whether upper classes continued to grow when resources were scarce and the overall population was losing numbers, and whether lower classes experienced relatively high mortality even when resources were relatively abundant and overall population growth continued. In both cases, the answer was yes.

We next conducted a detailed sensitivity analysis of the model. We varied the value of each of the key parameters incrementally up and down from the baseline values, running the simulation to see if altered parameter values would result in similar outputs with respect to relative population size, population stability, and rate of population extinctions for the different levels of inequality. See Supporting Information S1 for details of these tests.

We modified our simulation to allocate resources individual by individual according to a Pareto or Power Law Distribution, rather than by class. Three Pareto Index values were used, giving distributions with Gini coefficients of 0.14, 0.28, and 0.423. Each of these three distributions was generated randomly using the MatLab “gprnd” command [94]. As with the simulated class societies, actual allocation started with the wealthiest individual and moved down the list until resources ran out. Unlucky individuals at the bottom died of resource deprivation.

In order to compare the simulated dynamics of population instability in stratified populations to a simple analytical model, we constructed a simple recursive equation for logistic population growth, with a variance in carrying capacity—i.e. size of population that can be supported by available resources:
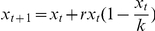
where x = population, t = time, r = population growth rate, and *k* = carrying capacity with a variance of ξ. The recursion was parameterized at six different levels of variance on *k* (equivalent to standard deviations from 16.6–33.3% of *k*), and iterated over 5000 time steps.

After using the above techniques to understand the basic simulation properties and outcomes, we altered the model to investigate the results of incorporating additional properties. We compared the following four situations for all 10 gradient levels: constant environment without storage (the baseline trials), constant environment with storage, variable environment without storage, and variable environment with storage. Storage consisted of saving some specified portion of “excess” resources (over the optimal 40 units) allocated to an individual in a given year, to be used later when less than the optimal amount of resources was available.

Variable environments were modeled by allowing three types of years – good years (ideal productivity rate according to the baseline model above), bad years (ideal productivity at 2/3rd the rate of good years) and very bad years (productivity at 1/3rd the rate of good years). We modeled this using a 3-state Markov model where each state corresponded to one of the three year types. Transition coefficients specified the probabilities of each state for the next year, based on the state in the current year.

We then ran trials in which populations could migrate as needed. The 100 sites were initialized with only 10 populations (5 egalitarian, and 5 stratified with inequality gradient 8), leaving 90 sites empty at the beginning. Populations were allowed to grow just as in the baseline trials, but also to migrate according to one of the following three migration triggers: declining population (specified threshold percent decline in total population numbers over 2 years at 8%, 13%, and 18%); resource deprivation (specified threshold amount of resources allocated per person for all egalitarians, and for individuals of the 4th class in stratified societies, at 36, 34, and 32); or resource depletion (specified threshold amount of resources remaining at a site at 45000, 30000, and 15000 total resource units).

In each case, when the threshold was reached, a total of 65 people (or half the remaining population for populations under 130) would attempt to migrate. If unoccupied sites were still available, migrating groups were assumed to reach their destination. When there were no unoccupied sites, migrating groups were assumed to die. Because populations went extinct from time to time, sites would periodically become available again for occupation by another group. For each trial we tracked how long it took for all 100 sites to fill, the number of migration events for each society type, the percent of populations going extinct for each society type, and the final fraction of sites held by egalitarian and stratified societies (see Supporting Information S1 for detailed results). After learning which threshold values for each type of migration trigger led to the highest fraction of sites occupied by egalitarian groups and by stratified groups, we ran new competition trials using this optimal trigger value for each type of society, tracking the same metrics as before.

## Supporting Information

Supporting Information S1 **Methods and Results.** Methodological details not provided in the text of the main article are found here, including explanations of the statistical tests used and the sensitivity analysis of the simulation, and the analysis of archaeological case studies. Extensive results for most parts of the study are also provided, using figures and tables for which there was not room in the main article.
(PDF)
