# Supplementary material for: The Spread of Inequality
Source: PLoS One. 2011 Sep 21;6(9):e24683. doi: 10.1371/journal.pone.0024683 (PMC3177824; doi:10.1371/journal.pone.0024683)
Supplement: Supporting Information S1 — Methods and Results. Methodological details not provided in the text of the main article are found here, including explanations of the statistical tests used and the sensitivity analysis of the simulation, and the analysis of archaeological case studies. Extensive results for most parts of the study are also provided, using figures and tables for which there was not room in the main article. (PDF) [file pone.0024683.s001.pdf]

Supporting Information For:

The Spread of Inequality

Deborah S. Rogers[1,2*], Omkar Deshpande[3] and Marcus W. Feldman[1]

[1] Department of Biology, Stanford University, Stanford, California, United States of America
[2] International Human Dimensions Programme, United Nations University, Bonn, Germany
[3] Department of Computer Science, Stanford University, Stanford, California, United States of America

* Email: rogers.deborah@gmail.com

METHODS AND RESULTS

Model Schematic

Figure S1 presents a schematic outline of the components of the model. Each time iteration in the simulation consists of one year, in which each of the operations shown in the schematic is undertaken.

Statistical Tests and Results for Baseline Trials

Model I Anova: stability by gradient level (1 - 10), where stability is defined as the percentage of time the population size at the site stays within ±5% around a mean population size measured within a sliding window of 100 years. Socioeconomic stratification significantly affected population stability in baseline simulation results ($p < .001$); see Figure S2 and Table S1. Egalitarian populations were much more stable in general, although with greater variance between the 100 different simulated populations. Egalitarian societies spent on average over 50% of the time (in 2000-year runs) in a state of stability (as defined), while stratified societies spent from about 1% to 7% of the time in a stable state, on average.

Model I Anova: demographic crises by gradient level (1 - 10). A demographic crisis or crash is defined as an event in which the population loses at least 25% of its size in one year or successive years of population decline. At no stage during a "crash" (as defined) will the population size at the site increase from one year to the next. Presence of socioeconomic stratification significantly affected the number of demographic crises in baseline simulation results ($p < .001$); see Figure S3 and Table S2. Egalitarian populations experienced fewer such population crises in general, averaging 3.9 per 2000-year run, while stratified societies averaged from 4.9 to 5.4 population crashes per 2000 years. Although significant, this is not a particularly large difference. However, stability captures the population dynamics better than this measure.

Kolmogorov-Smirnov Test: probability of extinction by gradient level (1 - 10). The probability of extinction is calculated as the total number of extinction events for the 100 populations over 2000 years, divided by 100. Presence of socioeconomic stratification significantly affected the probability of population extinctions in baseline simulation results ($p < .001$); see Figure S4 and Table S3. In these baseline trials with constant productivity and no migration, only populations at

higher levels of stratification (gradient levels 7, 8, 9 and 10) experienced extinctions, with a probability ranging from 1% to 3%.

Model I Anova: population responsiveness to carrying capacity by gradient level (1 - 10). This is defined as the ratio of population size to the carrying capacity (based on the total resources available). Presence of socioeconomic stratification significantly affected population responsiveness to resources and carrying capacity in baseline simulation results ($p < .001$); see Figure S5 and Table S4. Egalitarian populations averaged around 65% of carrying capacity, and frequently maintained their size at or just above 100% of carrying capacity for long periods of time (which coincided with the periods of stability as determined above). Stratified populations almost never maintained a close relationship with carrying capacity, but fluctuated up and down more or less continuously.

Model I Anova: resource depletion by gradient level (1 - 10). This is defined as the amount of time at a given site during which there is less than 75% of maximum total resources. Presence of socioeconomic stratification significantly affected resource depletion in baseline simulation results ($p < .001$); however, in the opposite direction than that expected; see Figure S6 and Table S5. It had been anticipated that stratified societies would deplete the resource base as effectively as egalitarians due to overuse of resources by wealthier classes. However, at the parameter values used in the baseline simulation, high mortality in lower classes over-rode this effect. Egalitarian populations had a much greater impact on the resource base in general, averaging over 75% resource depletion at least 50% of the time. Stratified populations, on the other hand, tended to deplete the resource base far less because these populations remained low due to high mortality in the lower classes.

Sensitivity Tests of Simulation Response to Parameter Values
The parameter values and results for each sensitivity test are shown in Figures S7 - S9 and Table S6, and in Figures S10 – S16 and Tables S7 – S11. Each table corresponds to a trial group in which we varied one of the parameters across a range of values. The values of the parameter are given in the first column (P). The rest of the columns show the values of various outcomes for egalitarians (E) and the nine inequality gradients for stratified populations (H2 - H10). The first row of each table corresponds to the baseline parameters, and the other rows correspond to trials with the parameter P systematically varying across the range of values. Results can be summarized as follows:

1. Egalitarian populations are always more stable at every parameter value except in trials when current resource amount determines ≥ .5 of the next year's productivity (see Figure S7). This appears to lead to a very unstable environment: once depletion begins, it exerts a positive feedback effect and causes a rapid crash of egalitarian populations.

2. Egalitarian populations always have fewer crashes except in trials at fertility = .135 (see Figure S8). This anomalous spot in parameter space seems to be just high enough to cause population growth and thus resource depletion, but too low to allow sufficient recovery.

3. Egalitarian populations are almost always much less likely to go extinct, except in trials when resource amount determines ≥ .5 of the next year's productivity (discussed in 1. above), and in trials at fertility > .18 (see Figure S9), which apparently causes egalitarian populations to grow

too large, thus depleting resources and resulting in rapid mortality. We varied the fraction of the resource increment that depends on the resources left from the past year (denoted as P in Table S6; baseline value = 1/3). Note that the last row of this table shows the only exception for which egalitarians have a slightly lower stability. However, this is an artificial parameter because when P becomes as high as 0.9, the resources of the site are renewed at an extremely low rate. They are almost the same as the resources remaining from the past year. This makes the resources at the site rapidly dwindle over successive years, causing rising mortality.

Comparison Trial Results

We compared four situations for all 10 gradient levels: constant environment without storage (the baseline trials), constant environment with storage, variable environment without storage, and variable environment with storage. Storage consisted of saving some specified portion, S, of "excess" resources (over the optimal 40 units) allocated to an individual in a given year, to be used later when less than the optimal amount of resources was available, as follows:

$$S = E\,(1 - 1/X)$$

where E is excess resources (allocated resources – 40) and X is a parameter we can vary. For example, if X = 10, 90% of the excess resource units (those beyond the 40 units that are needed for a healthy life) are stored. This annual storage was allowed to accumulate as long as excess resources were allocated (i.e. above 40 units). Because egalitarians were allocated only 40 resource units in the baseline trials, and thus would never have any excess to store, we increased their allocation to 76 resource units for these storage trials. Storage accrues to the entire group in the case of egalitarians, or to the entire class for stratified populations, rather than to individuals. When resources become limited (< 40 units per person), the resources accumulated over the years were allocated back to individuals in that class (or egalitarian group) equally, bringing total allocation per person up to a cap of 40 units.

Results of these comparisons are shown in Figure 2 in the article. These results show the following:

(1) Constant Environment without Storage: Egalitarians do far better: they have high levels of stability and no extinctions. Stratified societies have increasingly unstable populations and higher levels of extinctions as the inequality gradient increases.

(2) Constant Environment with Storage: Egalitarians with storage in a stable environment seem to have bimodal outcomes: either they crash and go extinct early on, or they make it through the initial crash and become completely stable from then on (storage no longer occurs because they run at population levels that prevent storage). Storage *per se* is not helpful on an ongoing basis—it may cause populations to exceed carrying capacity by a greater amount, which eventually leads to a harder crash. Stratified societies with storage seem to be protected slightly against extinctions, as compared with stratified societies in a stable environment without storage. In a stable environment, egalitarians without storage still do better than stratified societies with storage.

(3) Variable Environment without Storage: Egalitarians virtually always go extinct in an unstable environment, presumably because no effective behavioral responses to periodic resource

deprivation are allowed. Stratification (especially on the low end of the gradient) is protective against the instability, apparently because mortality is sequestered in the lower classes. Slightly stratified populations are somewhat unstable, but at least they survive.

(4) Variable Environment with Storage: Egalitarians are not protected by storage at the levels used in the trials. When we tried increasing storage, it did not help. Stratified societies are given additional protection by storage, although not much. In short, storage simply extends an unsustainable situation a bit longer—it doesn't ultimately alter the outcome.

Migration Trials

Results of the migration trials are presented in Figure 3, and Figures S17 – S20 and Tables S12 – S13. In the figures we distinguish between the "frontier" phase when unoccupied sites remain open for colonization, and the "carrying capacity" phase when all sites are filled and thus cannot be colonized (in our simulation) unless another population goes extinct. These results show that stratified societies migrate more, and are thus able to take over during the "frontier" phase of all six optimal trigger migration trials. During the "carrying capacity" phase in constant environments, the more stable egalitarian populations are able to occupy sites vacated by stratified group extinctions. In the real world, conflict and conquest would likely lead to continued expansion by stratified populations. During the "carrying capacity" phase in variable environments, more egalitarian population extinctions allow the stratified populations to continue to occupy a greater number of sites.

Alternative Models

As described in the Methods section of the main article, we developed an alternative model of inequality using a Pareto Distribution of resource allocation to individuals rather than allocation by class. Results of these trials, discussed in the Discussion section of the main article, are shown in Figures S21, S22 and S23.

As described in the Methods section of the main article, we also constructed a simple recursive equation for logistic population growth, with a variance in carrying capacity. Results of this approach, also discussed in the Discussion section of the main article, are shown in Figure S24.

Archaeological Case Studies Analysis

We compared predictions generated by our simulation with case studies on the early development of stratified societies in various parts of the world (see citations in main article). We selected studies that presented a clear time series of descriptions of the societies and mentioned relevant aspects such as population size, wealth inequalities, migration or conflict. For each case study, we asked whether the following indicators were mentioned in conjunction with the development of stratified societies:

1. Do stratified societies exhibit unequal allocation of resources (in addition to status differences)?
2. Do populations grow, overshoot carrying capacity, and then decline?
    2.5. Is this phenomenon more associated with stratified societies?
3. Do growing populations expand through migration to additional sites?
    3.5. Is this phenomenon more associated with stratified societies?
4. Is conflict associated with expansion to new sites, once the landscape is populated?
    4.5. Is this phenomenon more associated with stratified societies?

5. Are stratified societies associated with more variable environments?
6. Are stratified societies associated with more storage of surplus?
7. Did stratified societies increase in frequency through expansion and conquest (as opposed to internal change)?

For each of these indicators, we then assigned a code as follows:

0	no clear indication for or against (generally because it was not mentioned)
1	definitely observed
2	partially observed
3	partially contradicted
4	definitely contradicted

Our analysis is displayed as a matrix (see Table S14) with 15 rows (the 15 case studies or groups of studies) and 10 columns (the seven indicators and three sub-indicators listed above). Each cell contains the code assessing whether the studies tended to support or contradict our predictions.

###

Figure S1. Model schematic showing major components of the agent-based simulation. See text of manuscript and ESM for details.

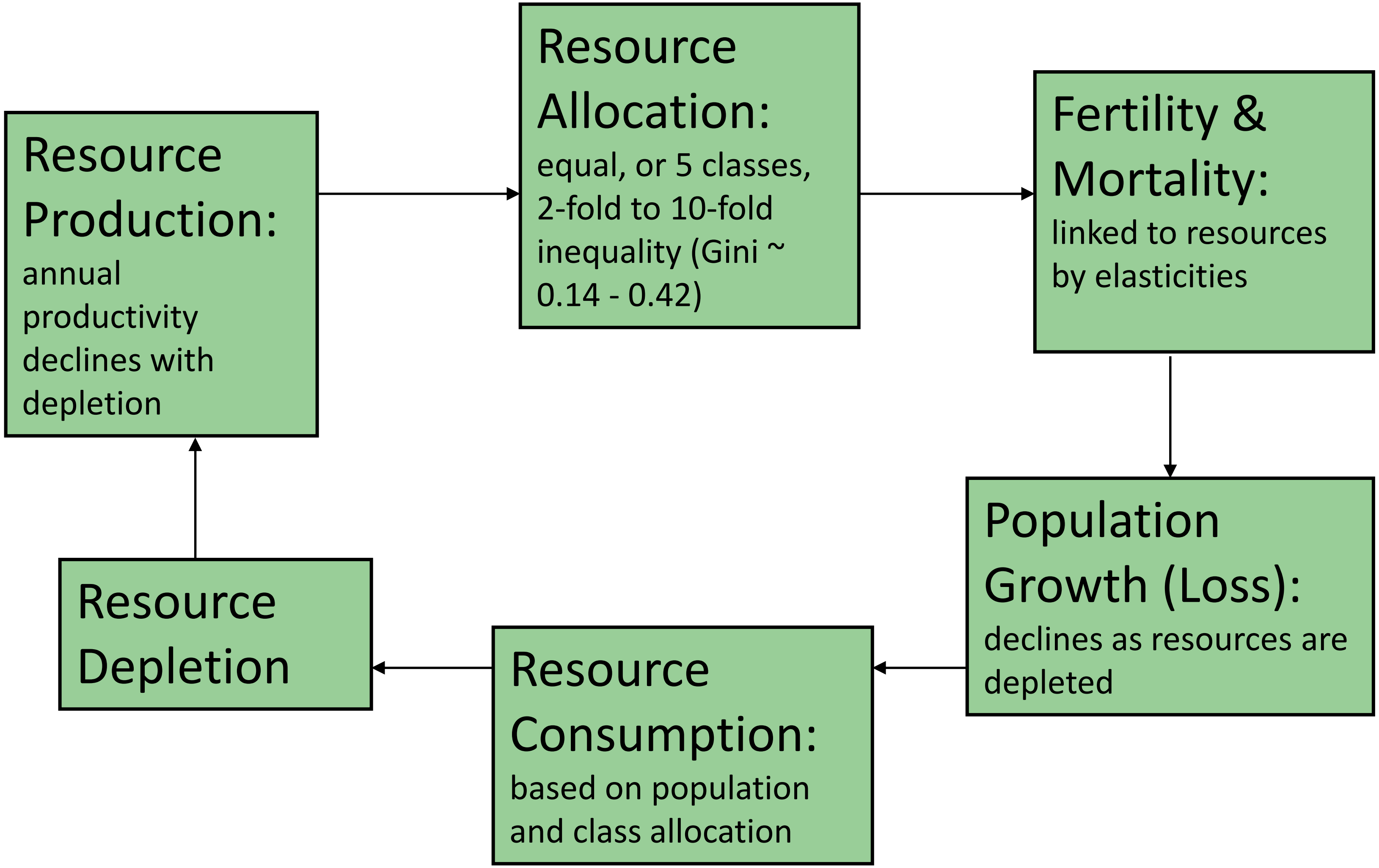

Figure S2. Population stability by inequality gradient level from 1 (egalitarians) to 10 (highly stratified). Stability is defined as the percentage of time the population size at the site stays within ±5% around a mean population size measured within a sliding window of 100 years. Differences between different levels of stratification are significant.

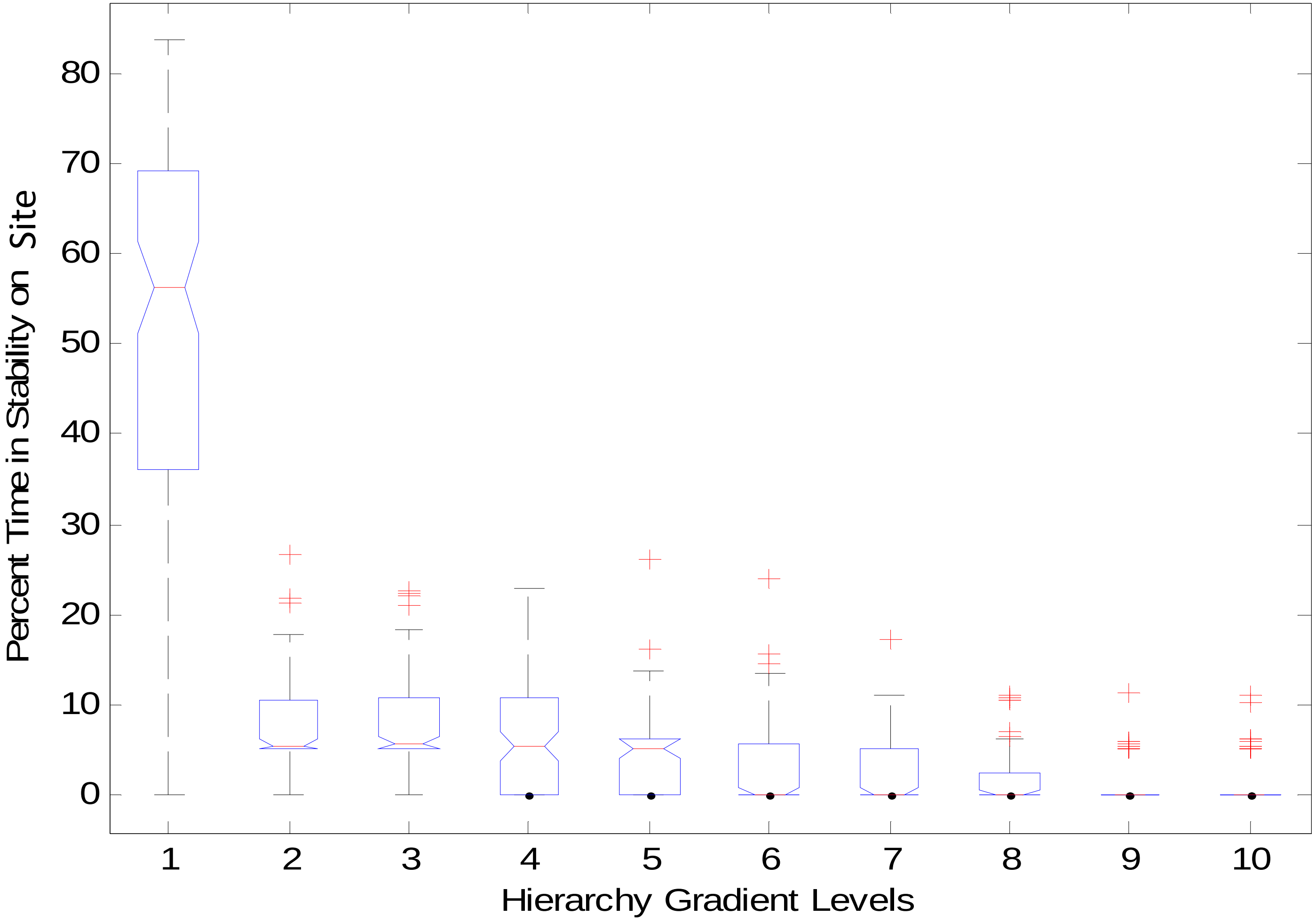

Figure S3. Demographic crises (over 2000 years) by inequality gradient by gradient level from 1 (egalitarians) to 10 (highly stratified). A demographic crisis or crash is defined as an event in which the population loses at least 25% of its size in one year or successive years of population decline. Differences between different levels of stratification are significant.

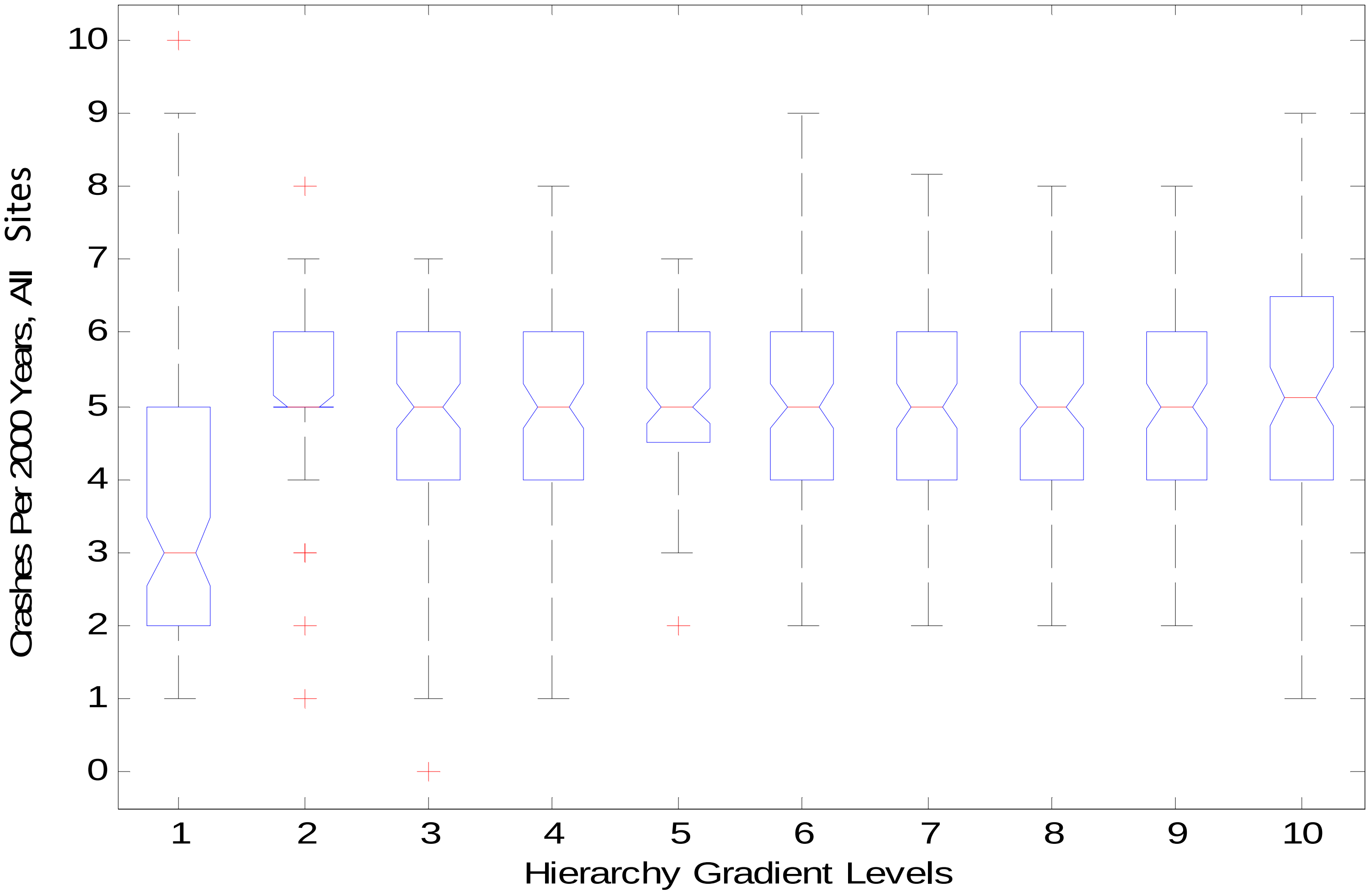

Figure S4. Probability of extinction by inequality gradient level from 1 (egalitarians) to 10 (highly stratified). Differences between different levels of stratification are significant.

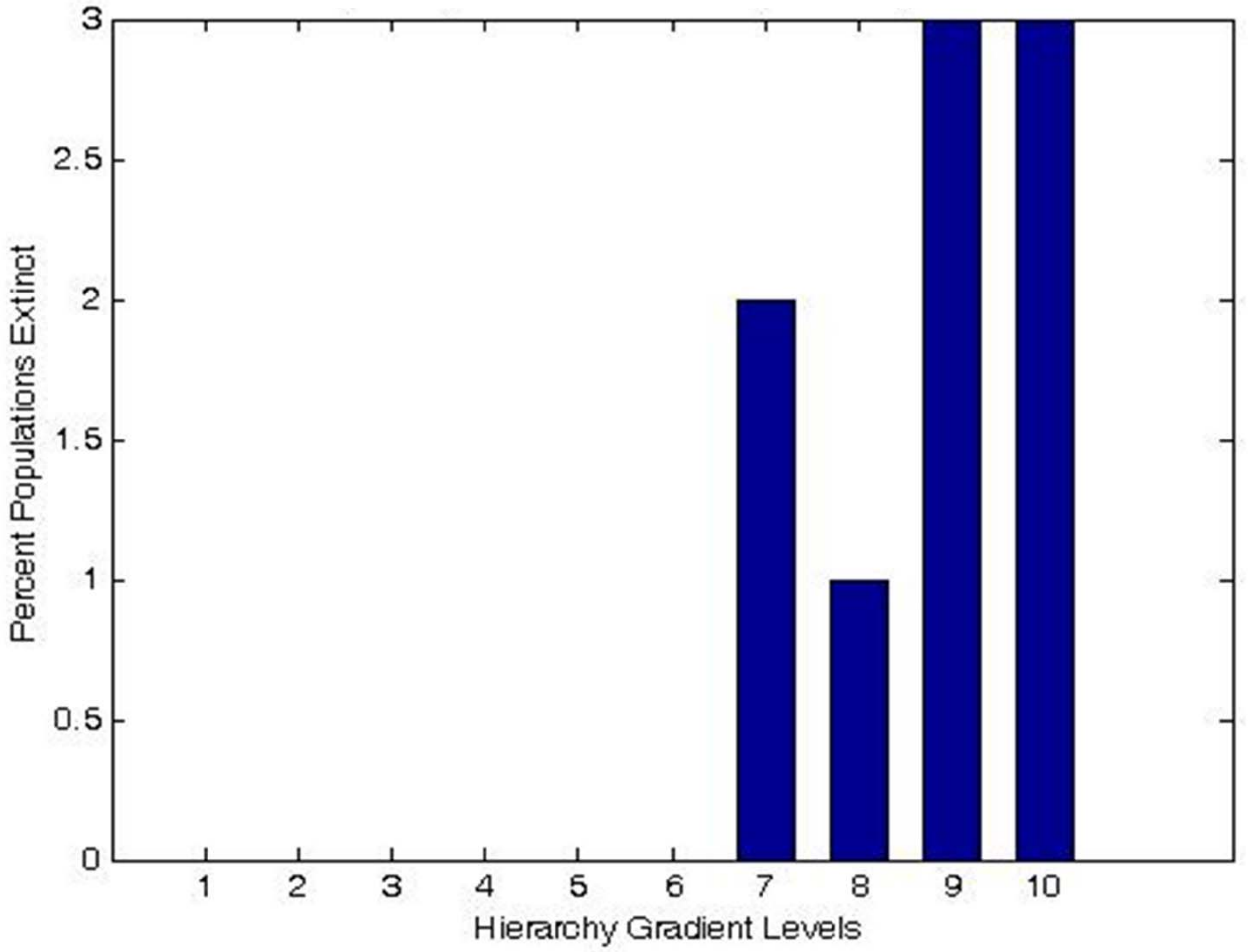

Figure S5. Responsiveness to carrying capacity by hierarchy gradient level from 1 (egalitarians) to 10 (highly stratified). Responsiveness is defined as the ratio of population size to the carrying capacity (number of people that could be supported based on the total resources available). Differences between different levels of stratification are significant.

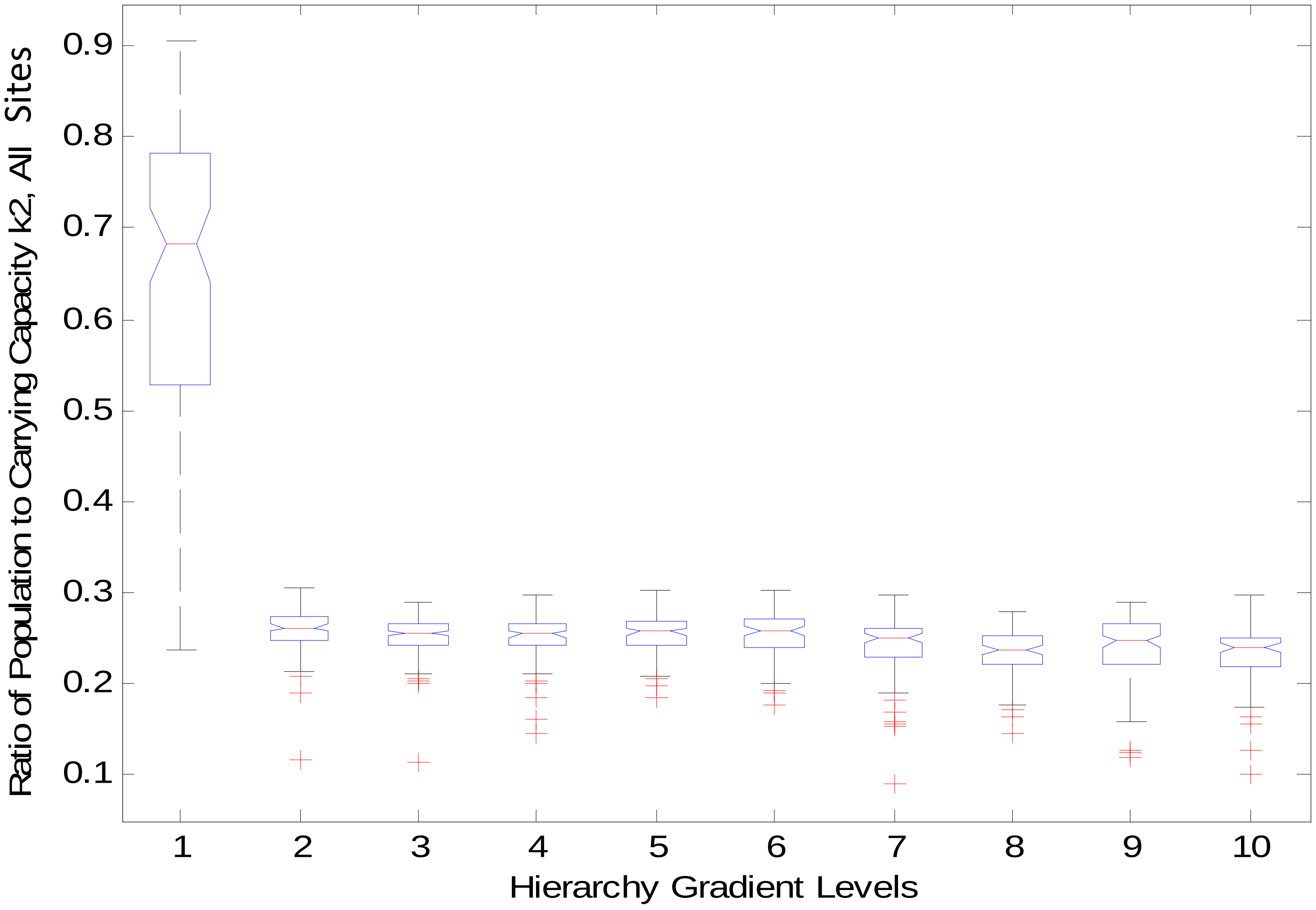

Figure S6. Resource depletion by hierarchy gradient level from 1 (egalitarians) to 10 (highly stratified). Resource depletion was defined as the amount of time at a given site during which there was less than 75% of maximum total resources. Differences between different levels of stratification are significant, but in the opposite direction from that which was hypothesized.

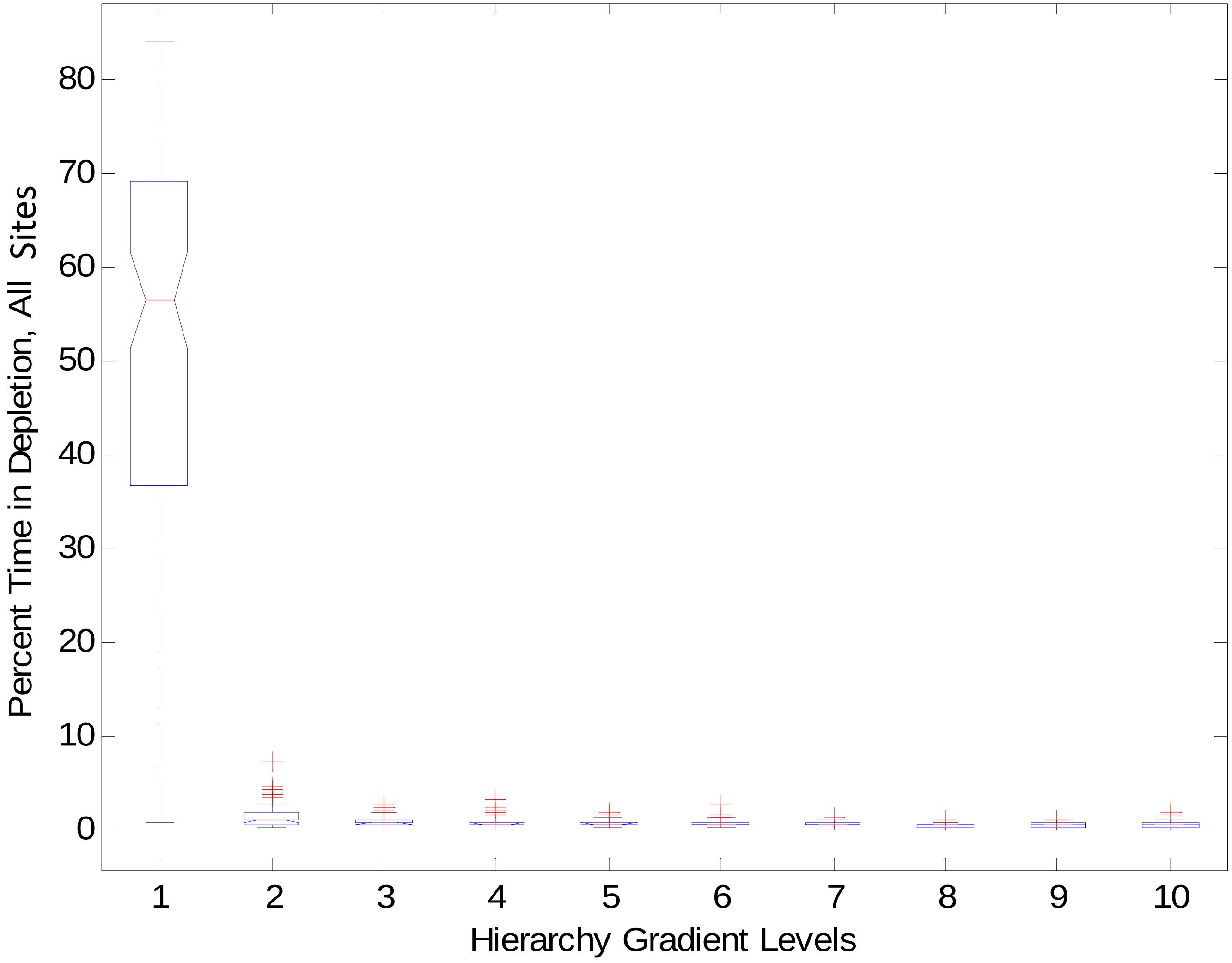

Figure S7. Sensitivity of extinction rate to the resource renewal function parameter value (fraction, S, of the resource increment that depends on the resources left from the past year). Egalitarian populations are almost always much less likely to go extinct, except in trials when standing resource amount determines ≥ .5 of the next year's productivity (shown here), and in trials at high fertility rates (see Figure S9). See ESM text for explanation.

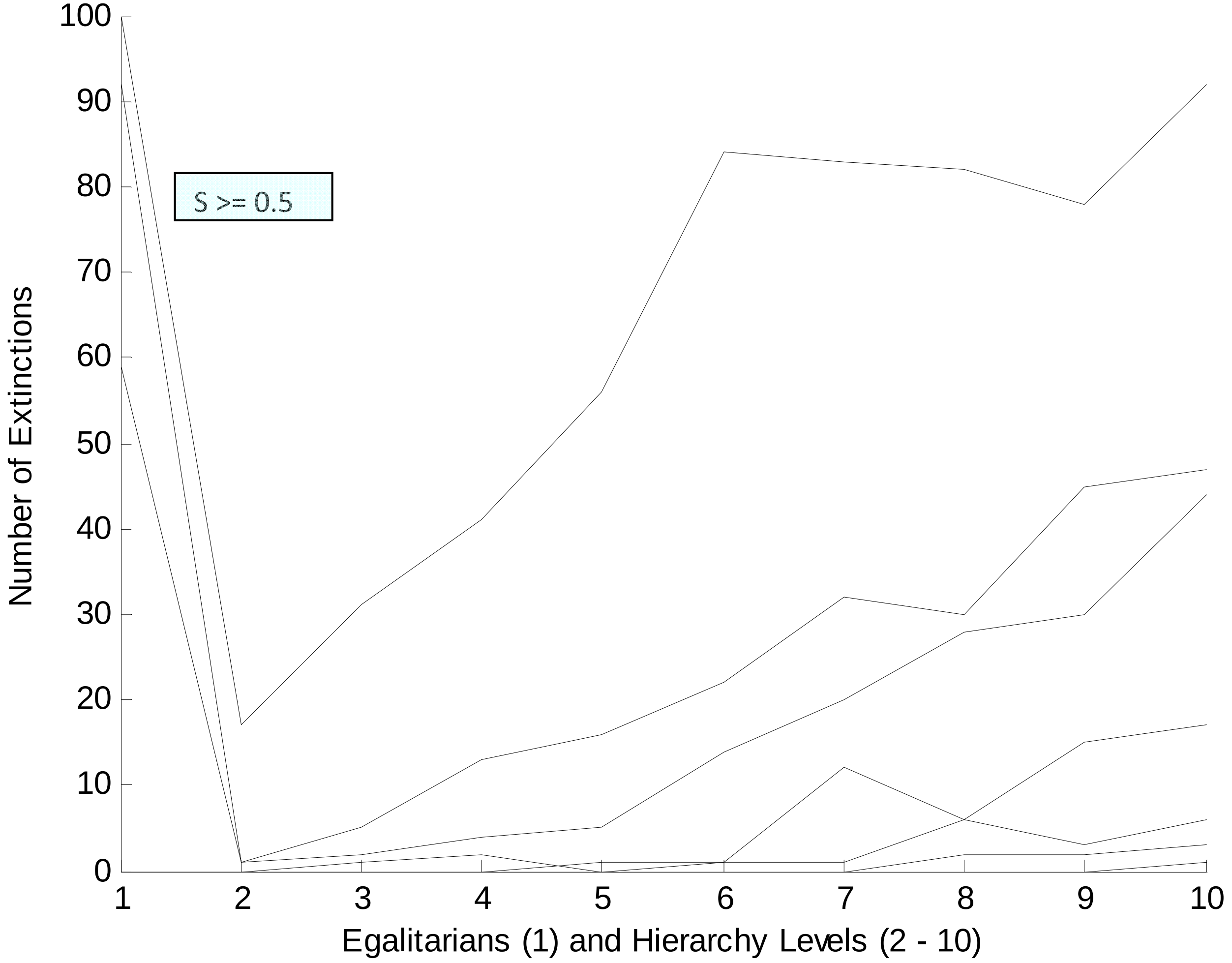

Figure S8. Sensitivity of demographic crises to maximum fertility rate parameter values. Egalitarian populations always have fewer crashes except in trials at fertility = .135. This anomalous spot in parameter space seems to be just high enough to cause population growth and thus resource depletion, but too low to allow sufficient recovery.

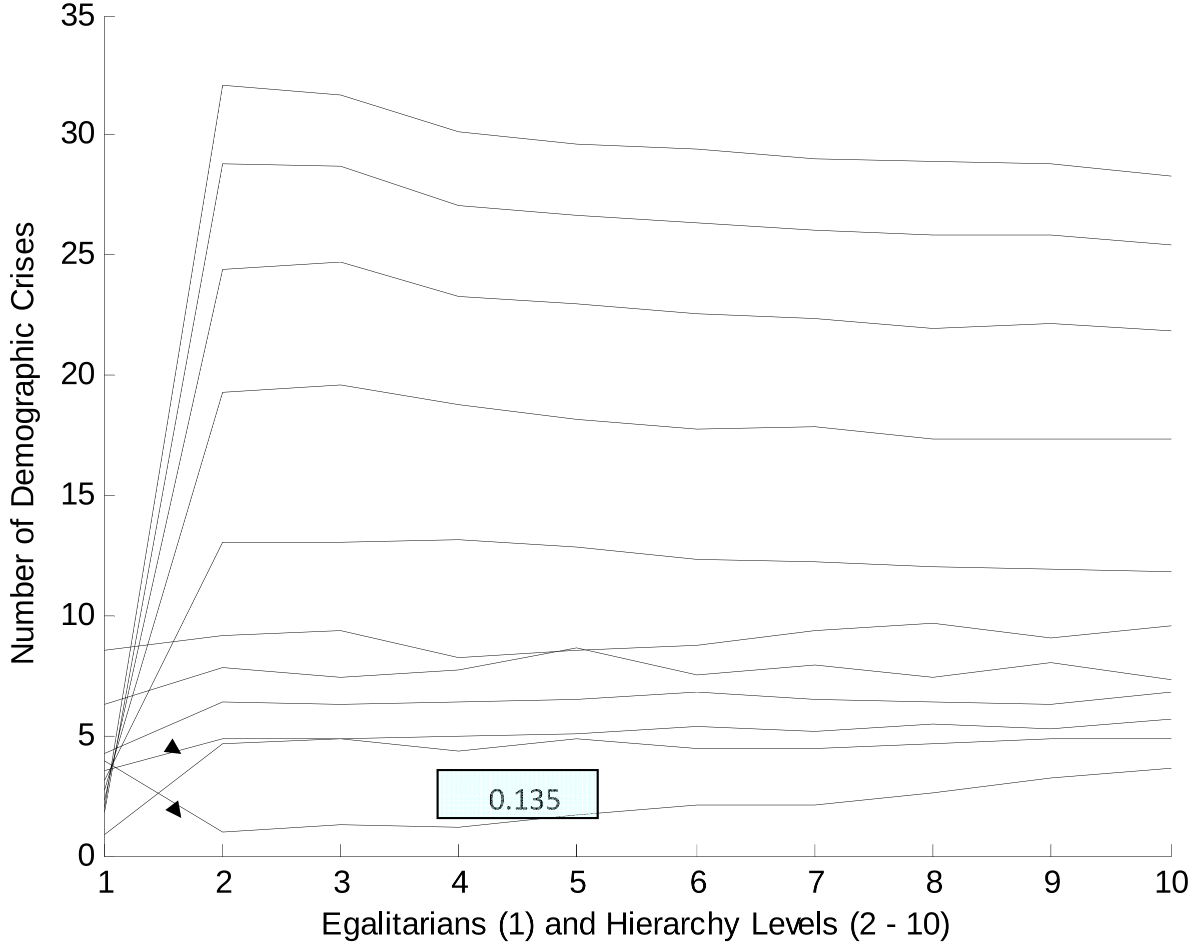

Figure S9. Sensitivity of extinctions to maximum fertility rate parameter values. Egalitarian populations are almost always much less likely to go extinct, except in trials when resource amount determines ≥ .5 of the next year's productivity (see Figure S7), and in trials at fertility > .18 (shown here), which apparently causes egalitarian populations to grow too large, thus depleting resources and resulting in rapid mortality.

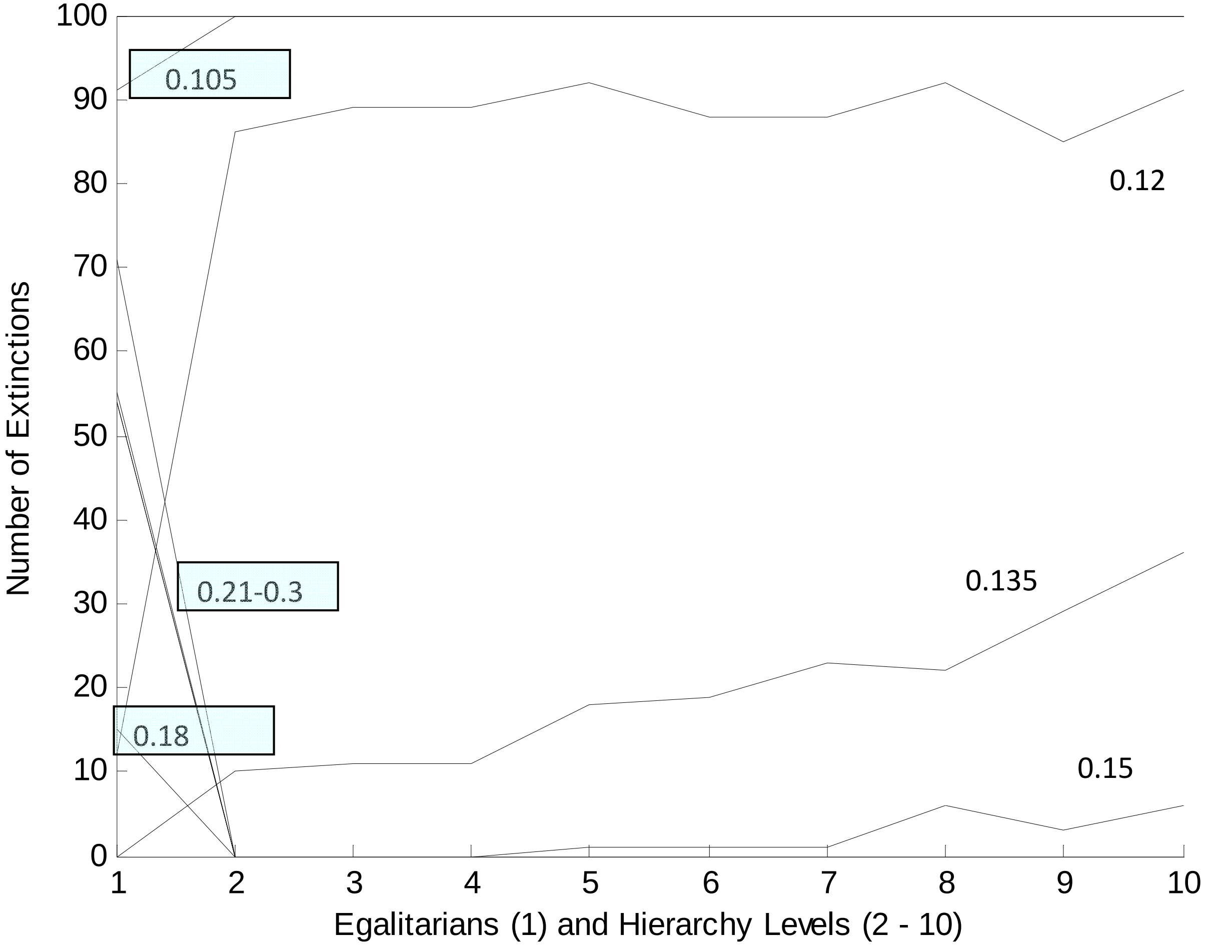

Figure S10. Sensitivity of population size, time spent in stability, number of demographic crises, and extinction rate to maximum fertility rate parameter values. See ESM text for explanations.

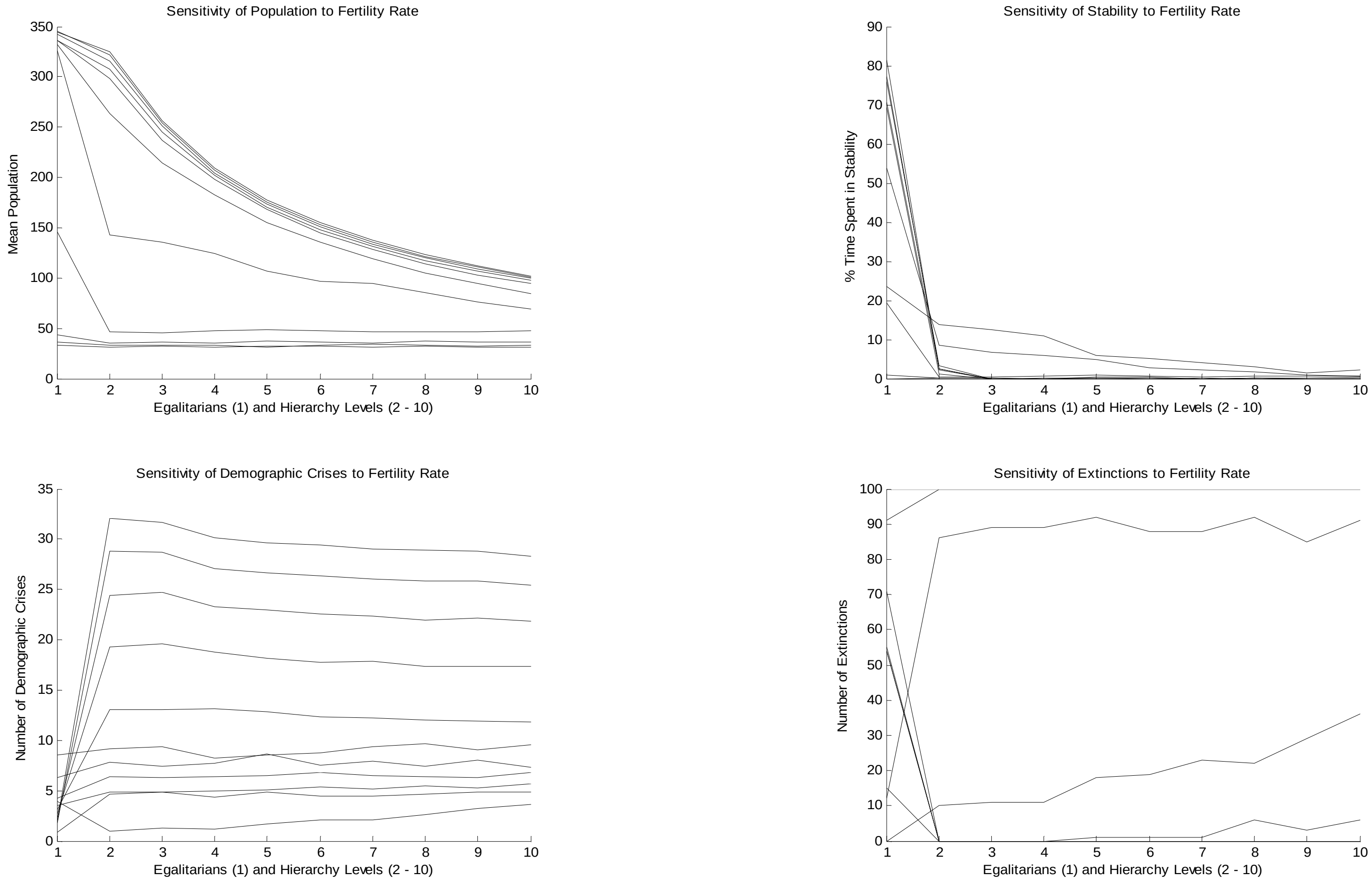

Figure S11. Sensitivity of population size, time spent in stability, number of demographic crises, and extinction rate to fertility resource cutoff parameter values. The fertility resource cutoff parameter was the lower end resource allocation below which women could not give birth.

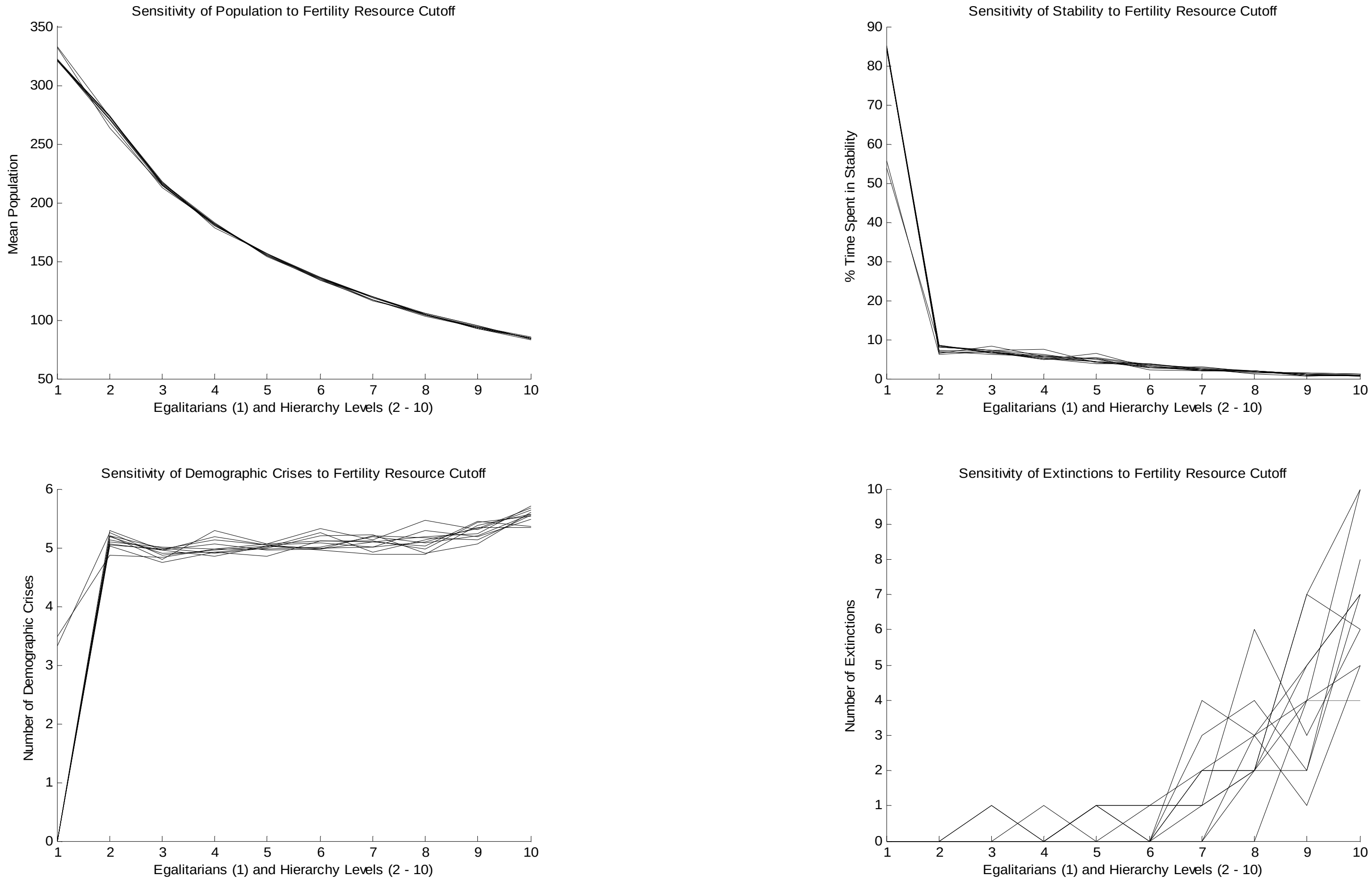

Figure S12. Sensitivity of population size, time spent in stability, number of demographic crises, and extinction rate to maximum survival rate parameter values. The maximum survival rate parameter is the highest survival rate given optimal resource allocation, which is then modified using an elasticity function linking actual resource allocation with survival, a class fitness metric, and an aging function).

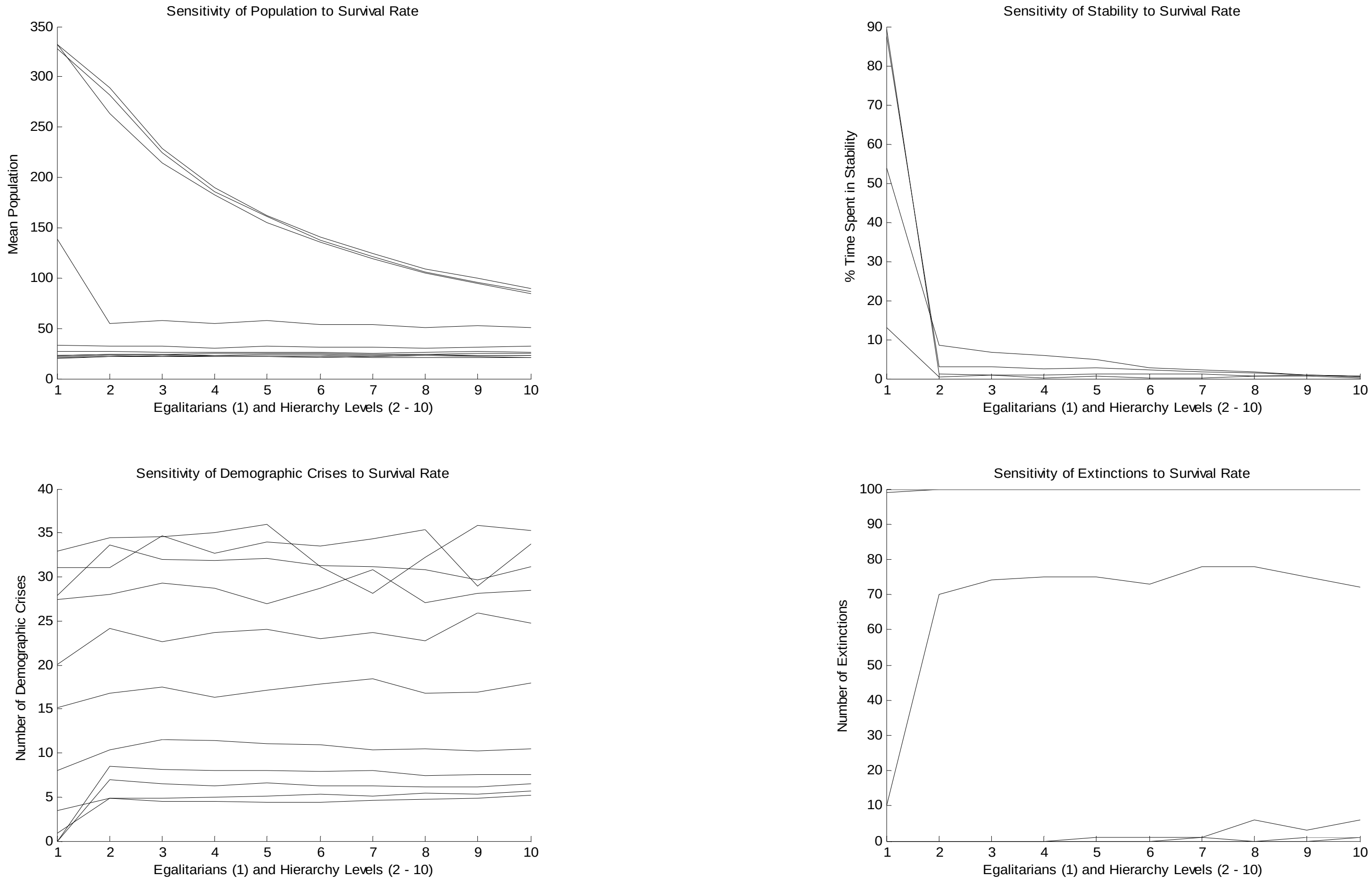

Figure S13. Sensitivity of population size, time spent in stability, number of demographic crises, and extinction rate to survival resource cutoff parameter values. The survival resource cutoff parameter was the lower end resource allocation below which no individual could survive.

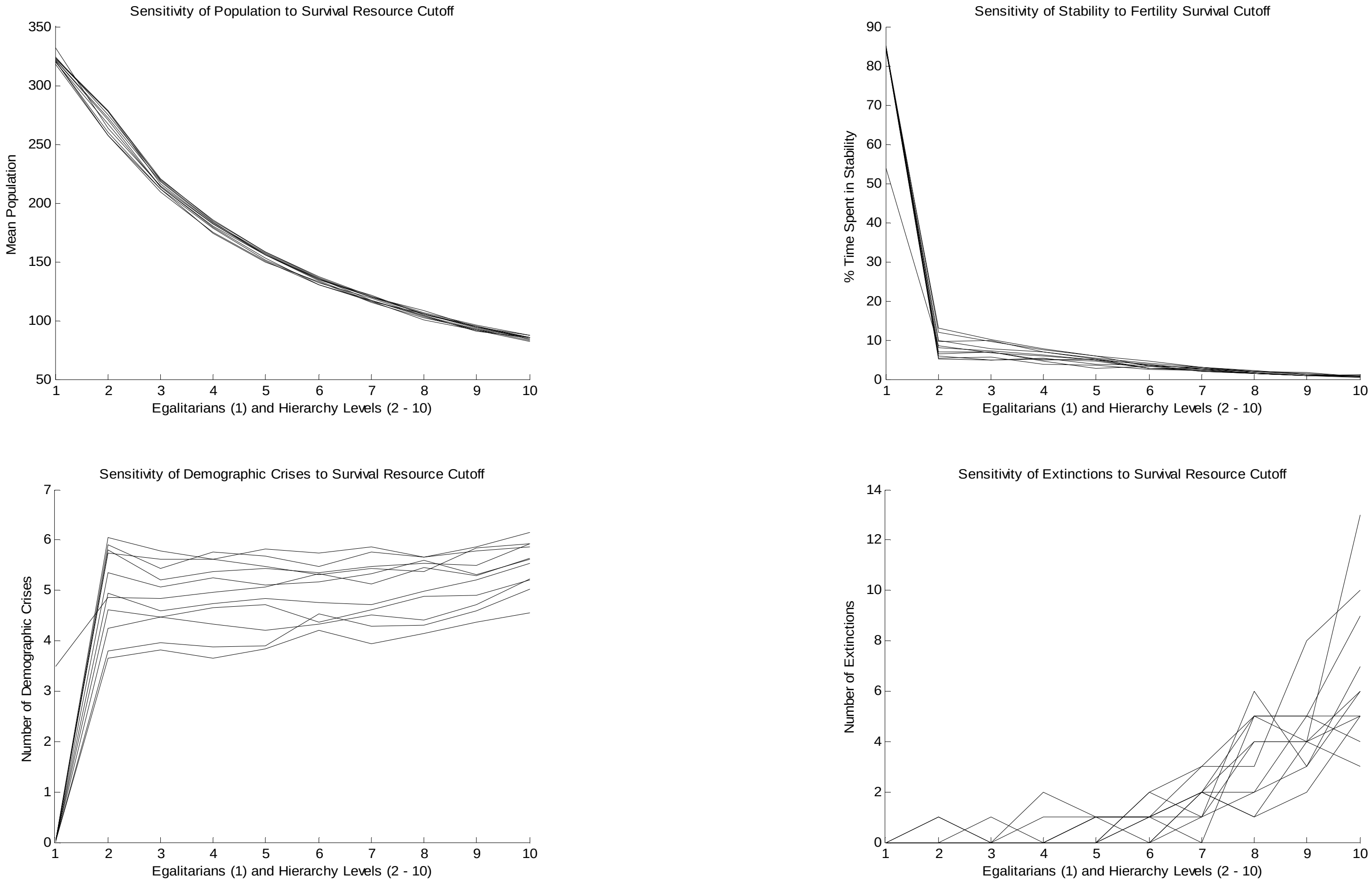

Figure S14. Sensitivity of population size, time spent in stability, number of demographic crises, and extinction rate to resource productivity parameter values. The resource productivity parameter gives the maximum rate at which resources would be renewed each year (applied in conjunction with a factor related to the amount of resource depletion that has taken place at that site—see ESM text for details).

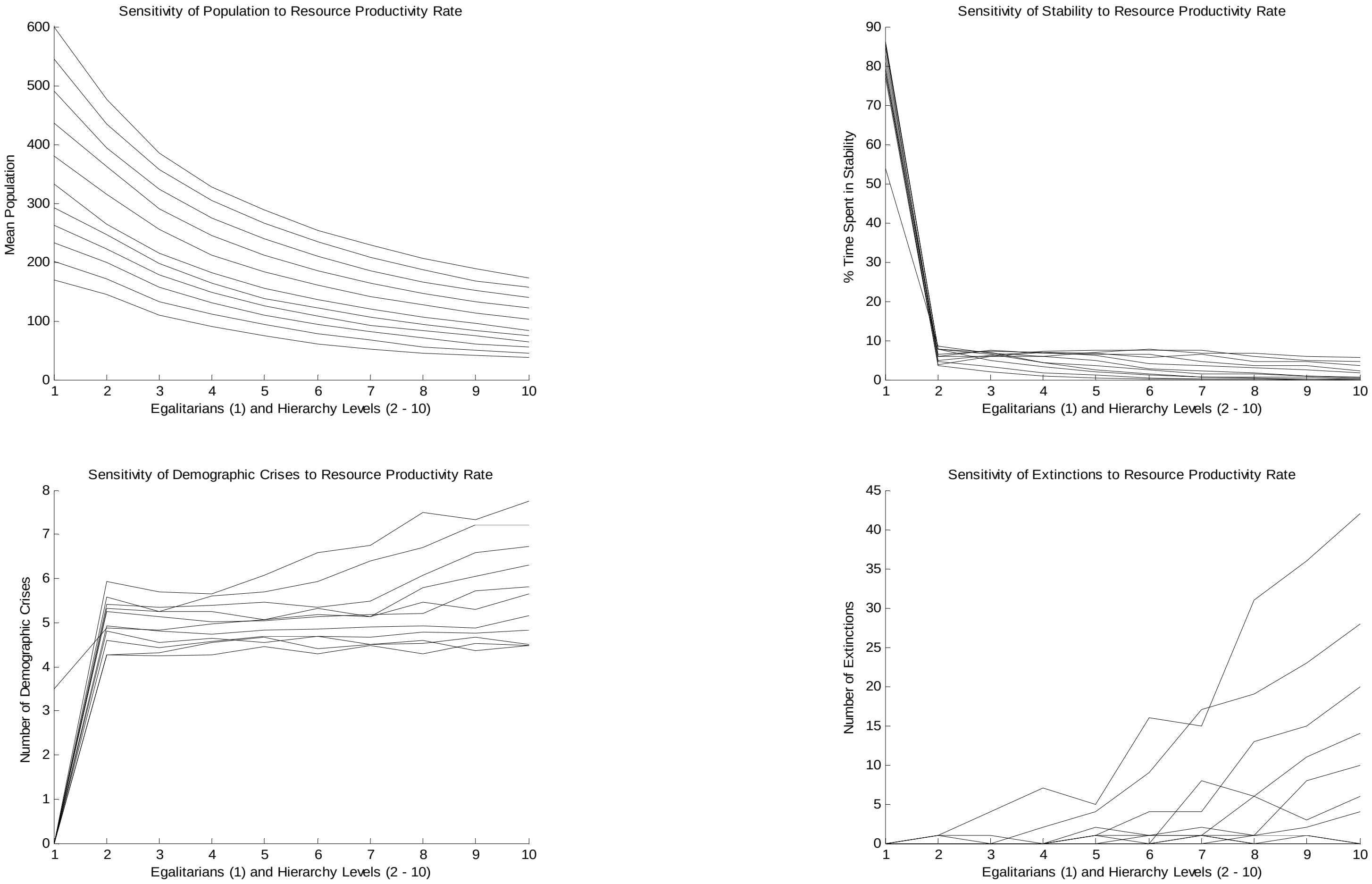

Figure S15. Sensitivity of population size, time spent in stability, number of demographic crises, and extinction rate to resource renewal function parameter values. The resource renewal function parameter expresses the extent to which actual resource production each year is related to current standing resource amount (or depletion), then applied in conjunction with maximum productivity rate (see ESM text for details).

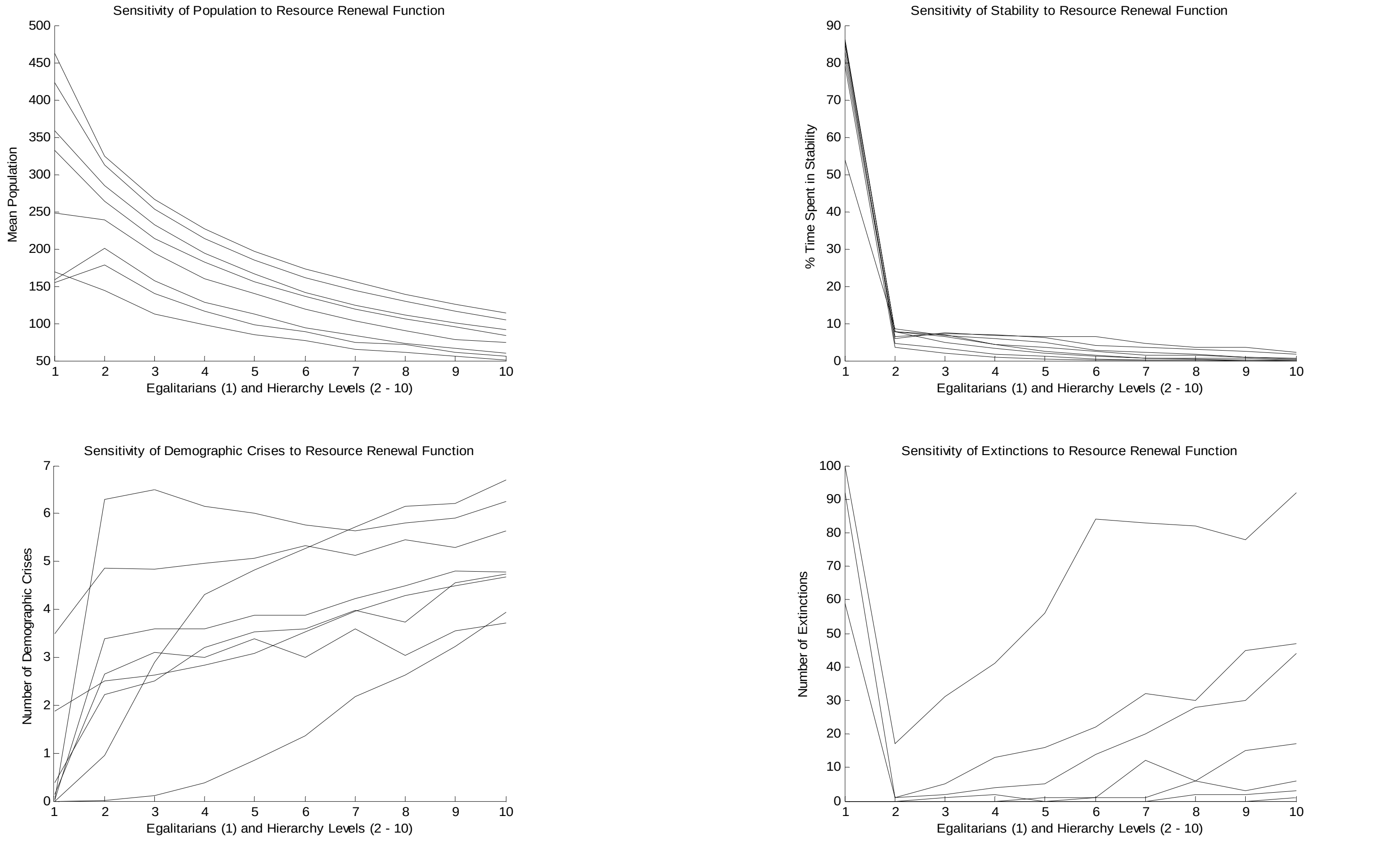

Figure S16. Sensitivity of time spent in stability to resource renewal function parameter values. Egalitarian populations are always more stable for every parameter value except in trials when current resource amount determines 0.9 of the next year's productivity, difficult to see here as the bottom curve which runs along or just above the x-axis. This appears to lead to a very unstable situation: once depletion begins, it exerts a positive feedback effect and causes a rapid crash of egalitarian populations. See also related Figure S7 on extinction rates.

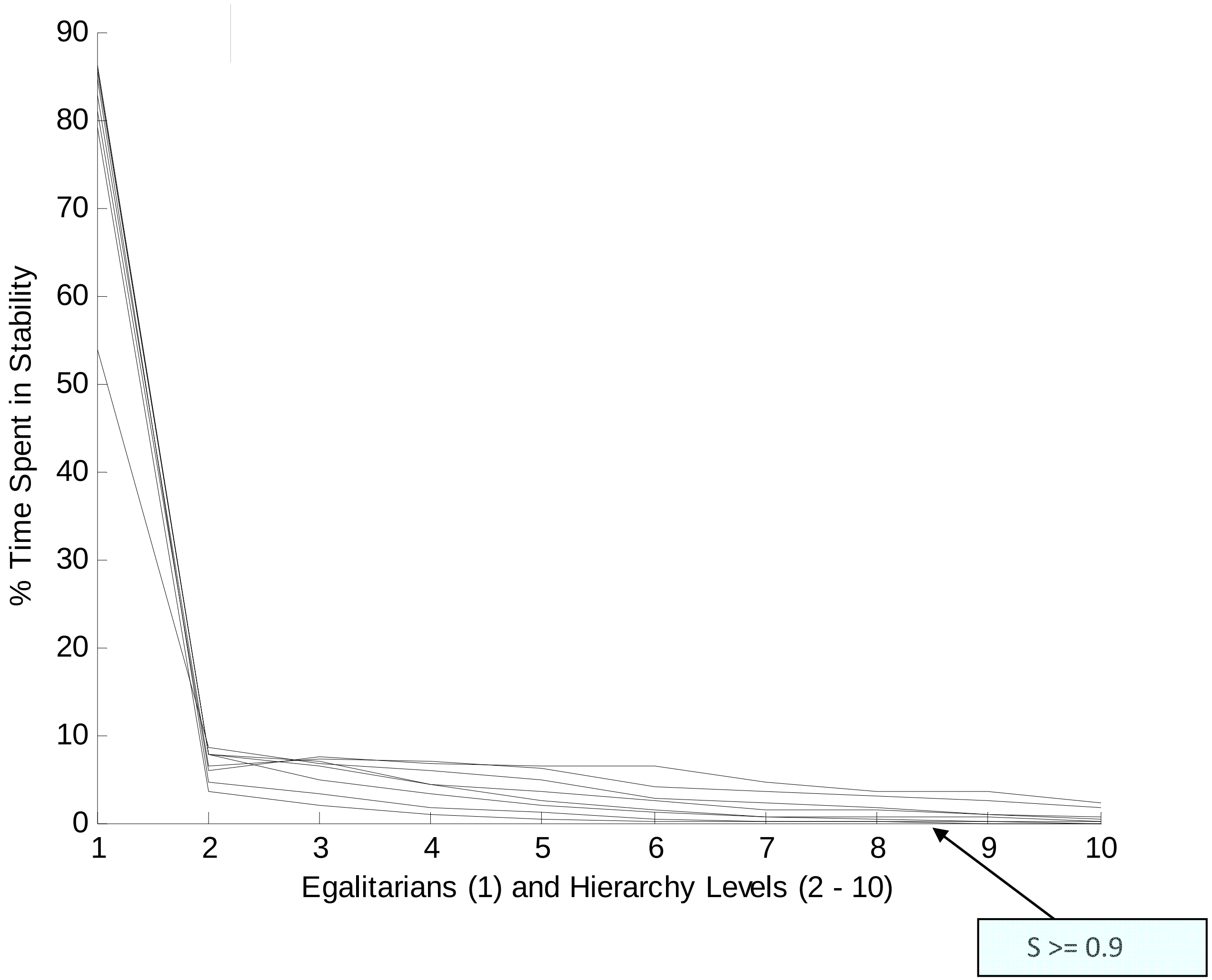

Figure S17. Migration competition, frontier phase, in constant environment, for 3 trigger types using optimal values for each population type. Frontier phase means the initial simulation years during which empty sites remain (shown by declining black line). The three trigger types are described in the ESM text. Stratified populations always migrate more and thus take over sites more rapidly, despite having smaller, more extinction-prone populations.

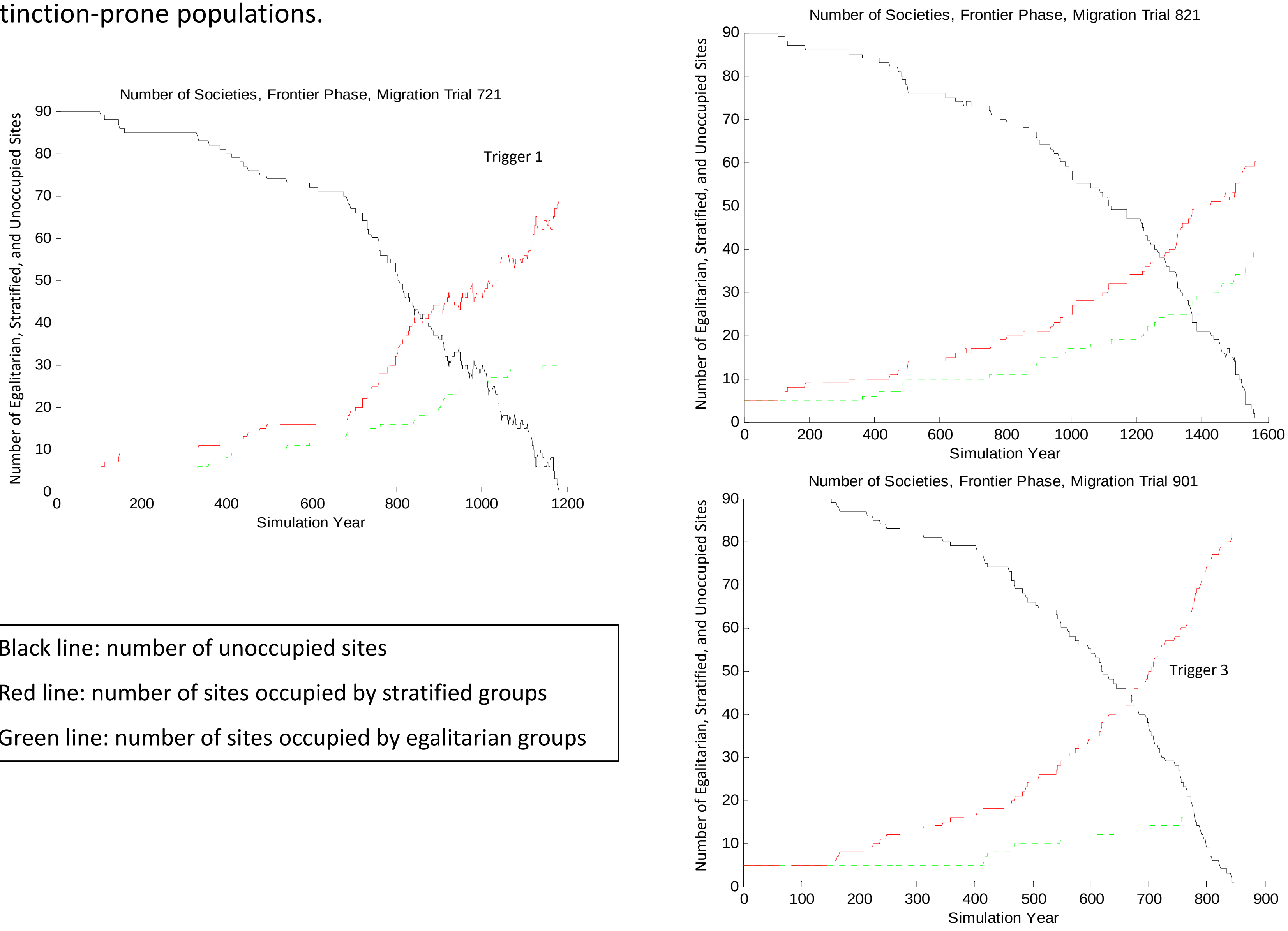

Figure S18. Migration competition, carrying capacity phase, in constant environment, for 3 trigger types using optimal values for each population type. Carrying capacity phase means the later simulation years during which sites are generally occupied but periodically open up due to population extinctions (shown by spiking black line at bottom). The three trigger types are described in the ESM text. For two of the trigger types, stratified populations gradually lose their advantage in number of sites, as they are more extinction-prone. The third trigger, total resource depletion at site, allowed stratified populations to avoid high extinction rates and thus maintain their advantage over the 10,000-year span of the competition trial.

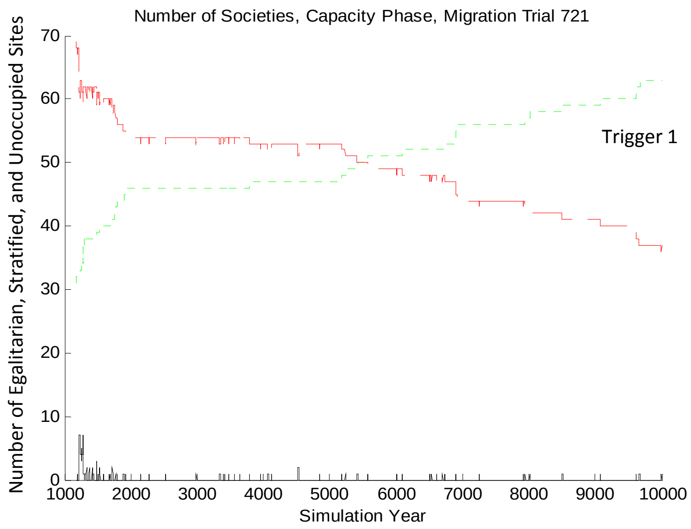

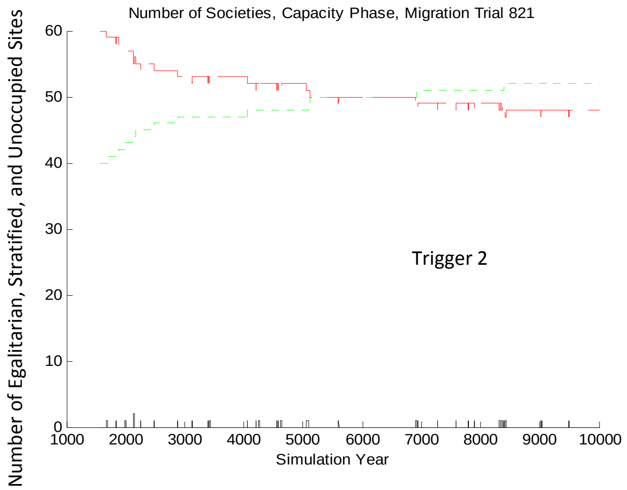

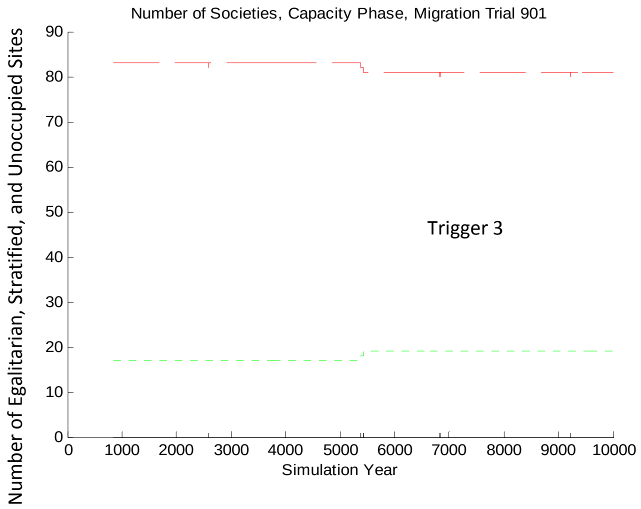

Black line: number of unoccupied sites

Red line: number of sites occupied by stratified groups

Green line: number of sites occupied by egalitarian groups

Figure S19. Migration competition, frontier phase, in variable environment, for 3 trigger types using optimal values for each population type. Frontier phase means the initial simulation years during which empty sites remain (shown by declining black line). The three trigger types are described in the ESM text. Stratified populations always migrate more and thus take over sites more rapidly, despite having smaller, more extinction-prone populations.

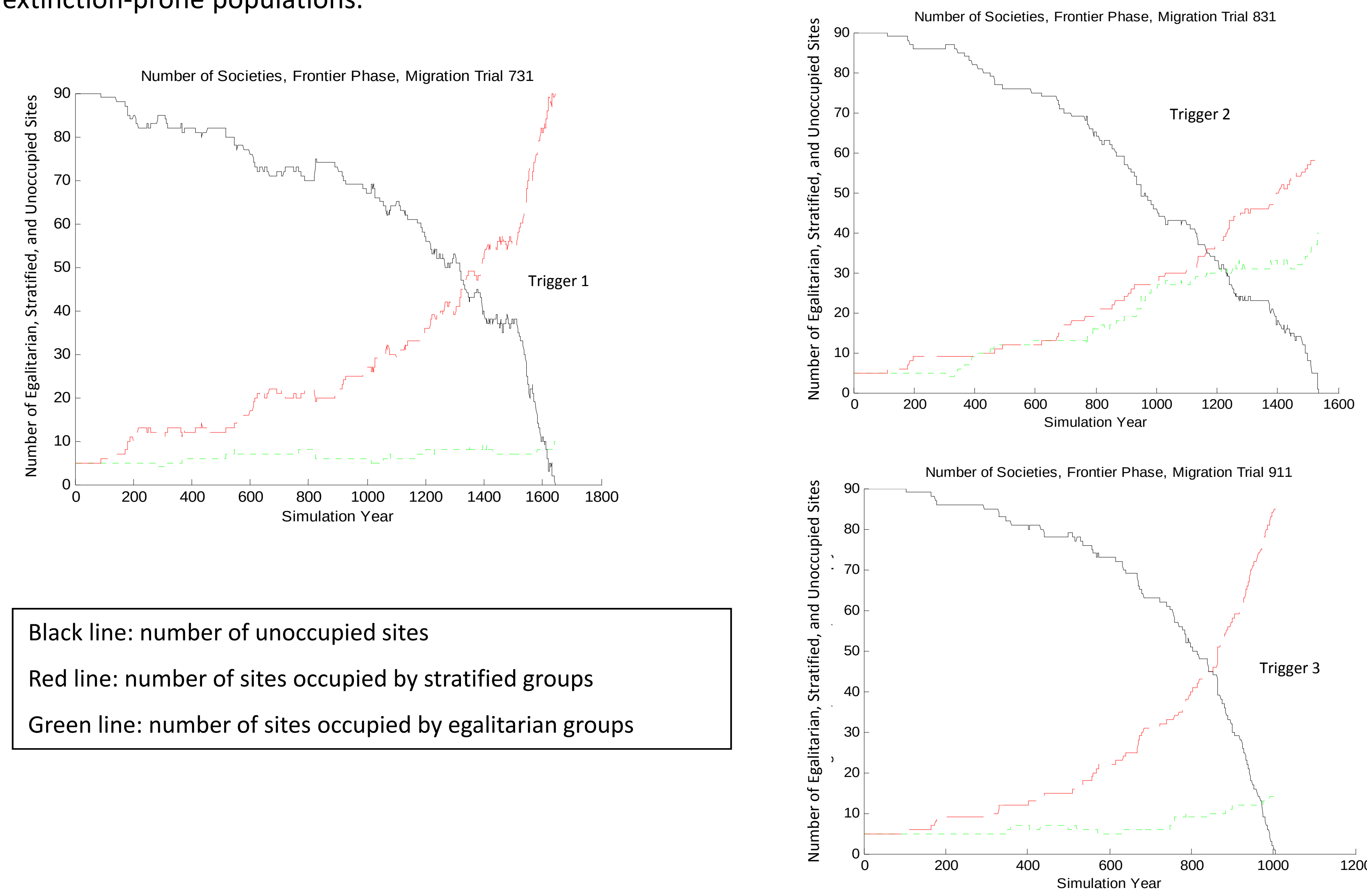

Black line: number of unoccupied sites

Red line: number of sites occupied by stratified groups

Green line: number of sites occupied by egalitarian groups

Figure S20. Migration competition, carrying capacity phase, in variable environment, for 3 trigger types using optimal values for each population type. Carrying capacity phase means the later simulation years during which sites are generally occupied but periodically open up due to population extinctions (shown by spiking black line at bottom). The three trigger types are described in the ESM text. For all three trigger types, stratified populations maintain the advantage gained through rapid initial migration to sites because they have a lower extinction rate in variable environments than do egalitarian populations.

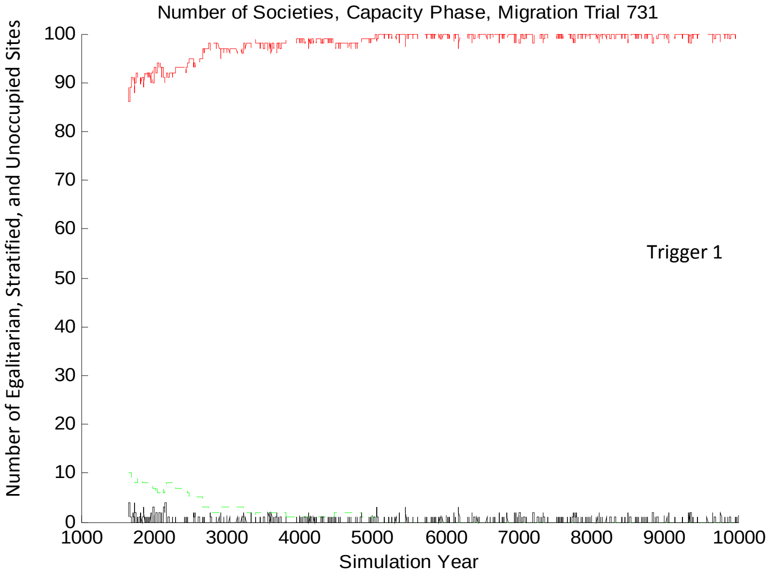

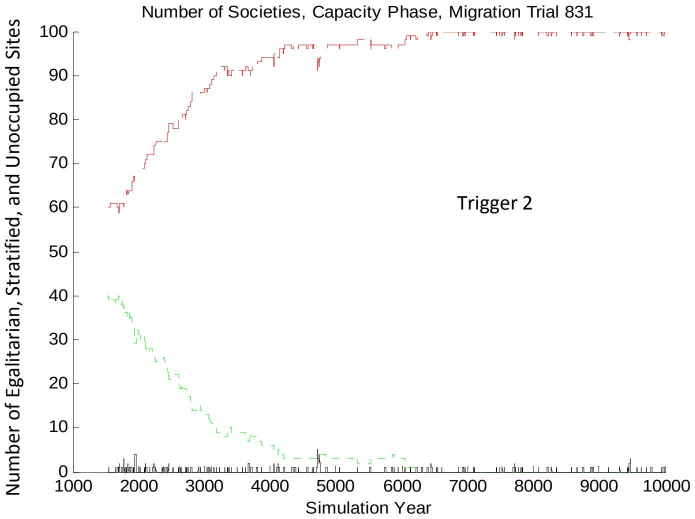

Black line: number of unoccupied sites

Red line: number of sites occupied by stratified groups

Green line: number of sites occupied by egalitarian groups

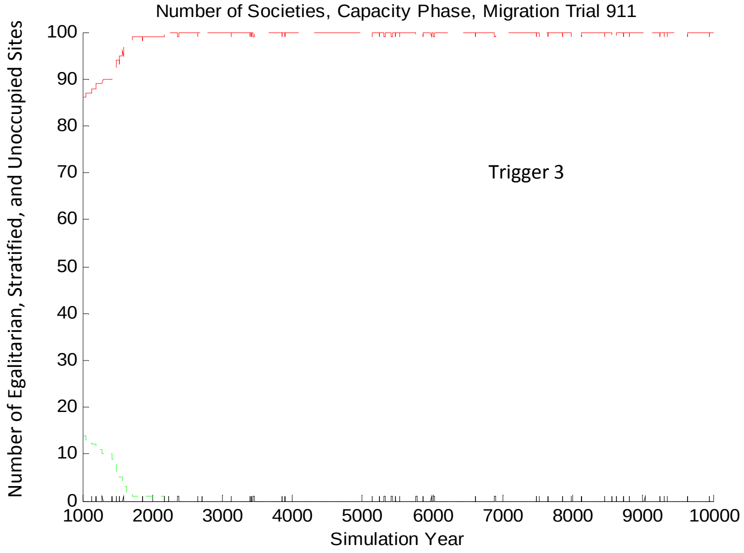

Figure S21. Typical populations with Pareto distribution of resources at three levels of inequality. As with populations stratified into five classes, these Pareto distribution populations show increasingly irregular population variability over time as the level of inequality becomes more extreme.

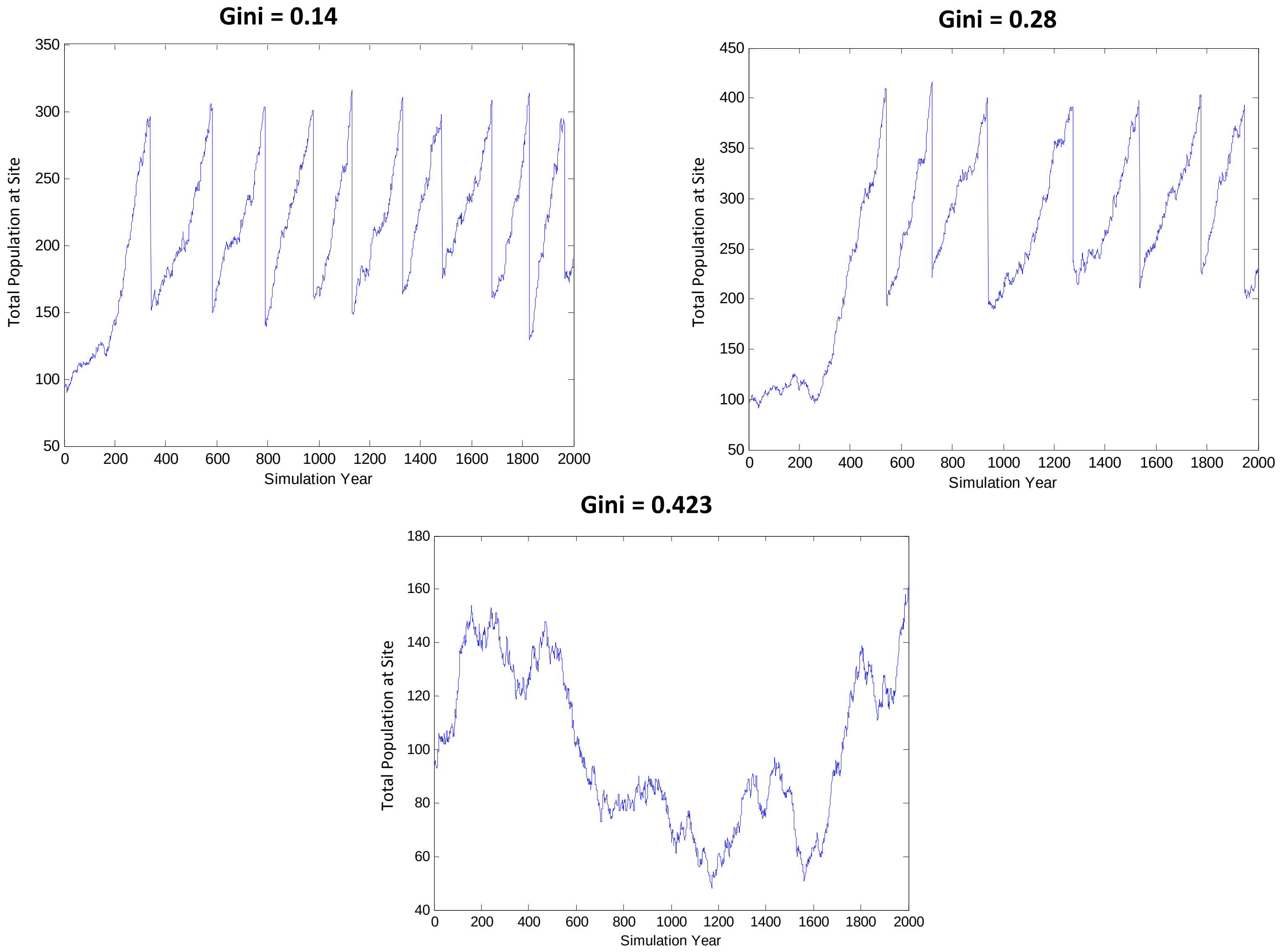

Figure S22. Percent time spent in stability for populations with Pareto distribution of resources at three levels of inequality. Stability is defined as the percentage of time the population size at the site stays within ±5% around a mean population size measured within a sliding window of 100 years. As with populations stratified into five classes, these Pareto distribution populations are less stable over time than are the egalitarian populations.

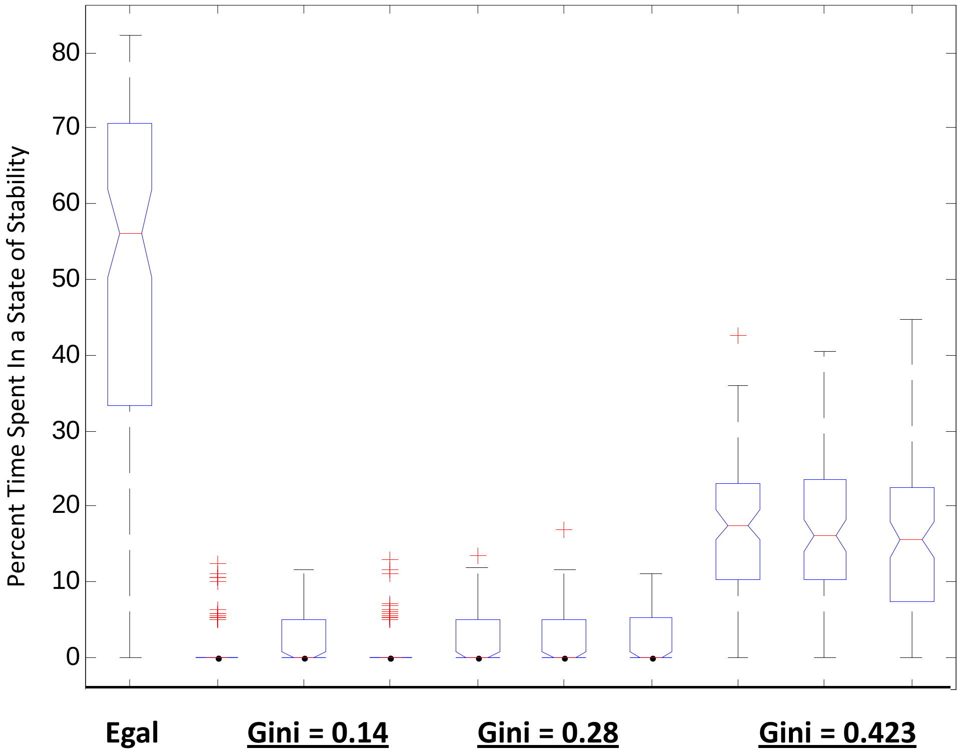

Figure S23. Number of demographic crises or crashes for populations with Pareto distribution of resources at three levels of inequality. A demographic crisis or crash is defined as an event in which the population loses at least 25% of its size in one year or successive years of population decline. As with populations stratified into five classes, two of these Pareto distribution populations experience significantly more crashes than did the egalitarian populations. The exception occurred in populations with the most extreme Pareto index: although highly unstable, these populations were so small that they rarely exceeded carrying capacity and crashed.

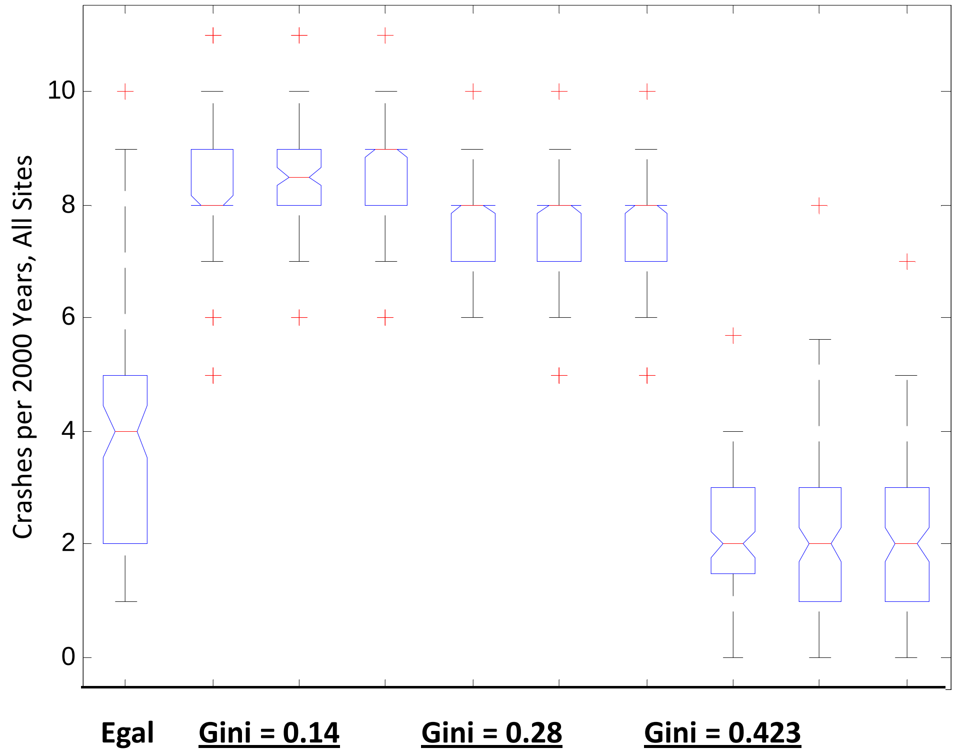

Figure S24. Population trajectory for different values of carrying capacity variance. As variance of the carrying capacity increased, the trajectory of the population over time became increasingly irregular, and eventually began to generate population extinctions, just as with increasing the resource allocation inequality gradient for stratified societies

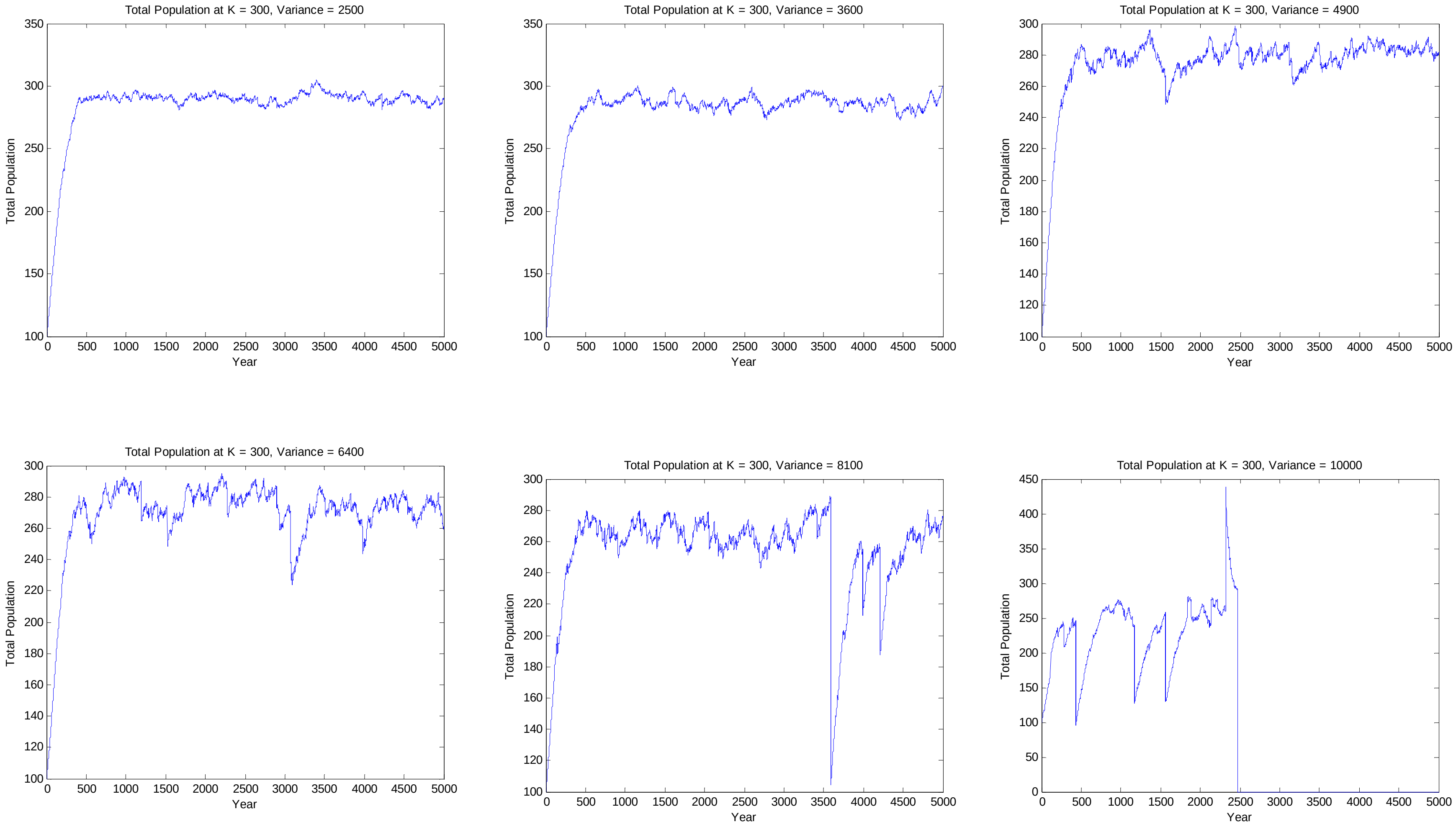

Table S1. Model I Anova on time spent with stable population by gradient level from 1 (egalitarians) to 10 (highly stratified). Stability is defined as the percentage of time the population size at the site stays within ±5% around a mean population size measured within a sliding window of 100 years. Differences between different levels of stratification are significant.

**Source**	**SS**	**Df**	**MS**	**F**	**Prob>F**
**Columns**	206101.4	9	22900.15	338.7612	0
**Error**	66923.69	990	67.59969		
**Total**	273025.1	999			

Table S2. Model I Anova on numbers of demographic crises (over the 2000 years) by gradient level from 1 (egalitarians) to 10 (highly stratified). A demographic crisis or crash is defined as an event in which the population loses at least 25% of its size in one year or successive years of population decline. Differences between different levels of stratification are significant.

**Source**	**SS**	**Df**	**MS**	**F**	**Prob>F**
**Columns**	170.0793	9	18.89771	9.0434	3.22E-13
**Error**	2068.771	990	2.089668		
**Total**	2238.851	999			

Table S3. Kolmogorov-Smirnov Test on probability of extinction by gradient level from 1 (egalitarians) to 10 (highly stratified). Differences between different levels of stratification are significant.

**TRIAL**	1
**Reject Ho?**	Yes
**P =**	0
**K-S statistic**	9.999631
**cutoff value**	0.133376

Table S4. Model I Anova on population responsiveness to carrying capacity by gradient level from 1 (egalitarians) to 10 (highly stratified). Responsiveness is defined as the ratio of population size to the carrying capacity (number of people that could be supported based on the total resources available). Differences between different levels of stratification are significant.

Source	SS	df	MS	F	Prob>F
**Columns**	14.64752	9	1.627502	453.1252	0
**Error**	3.555809	990	0.003592		
**Total**	18.20332	999			

Table S5. Model I Anova on resource depletion by gradient level from 1 (egalitarians) to 10 (highly stratified). Resource depletion was defined as the amount of time at a given site during which there was less than 75% of maximum total resources. Differences between different levels of stratification are significant, but in the opposite direction from that which was hypothesized.

**Source**	**SS**	**df**	**MS**	**F**	**Prob>F**
**Columns**	232537.4	9	25837.49	528.6591	0
**Error**	48384.89	990	48.87363		
**Total**	280922.3	999			

Table S6. Population stability for different values of the resource productivity function parameter. The first row of each table corresponds to the baseline parameters, and the other rows correspond to trials with the parameter P systematically varying across its feasible range. Results are shown for egalitarian (E) and all nine stratified (H) population types.

P	E	H2	H3	H4	H5	H6	H7	H8	H9	H10
0.333	53.839	8.67	6.751	5.792	4.9765	2.8567	2.199	1.79	1.053	0.606
0	80.054	33.042	13.78	7.837	6.5715	5.078	3.842	2.505	2.383	1.239
0.1	81.721	13.485	8.214	7.13	4.84	6.3375	3.095	3.089	2.382	1.638
0.25	83.656	6.83	5.87	8.102	4.816	4.024	2.019	1.764	1.852	1.147
0.5	84.81	6.951	6.389	4.448	4.0005	2.022	1.935	1.112	0.602	0.597
0.666	38.157	6.4855	4.488	2.602	2.3392	1.1123	0.914	0.616	0.595	0.668
0.75	17.166	3.9697	3.967	2.549	2.3259	1.544	1.394	1.074	0.817	0.346
0.9	0	3.7825	2.433	2.768	1.8878	1.5758	0.94	1.237	0.567	0.496

Table S7. Population stability for different values of the parameter 'maximum fertility probability.' The first row of each table corresponds to the baseline parameters, and the other rows correspond to trials with the parameter (P) systematically varying across its feasible range. Results are shown for egalitarian (E) and all nine stratified (H) population types.

P	E	H2	H3	H4	H5	H6	H7	H8	H9	H10
0.15	53.839	8.67	6.751	5.792	4.9765	2.8567	2.199	1.79	1.053	0.606
0.18	69.431	1.311	0	0.052	0.1035	0.05	0	0.055	0	0.11
0.21	70.597	2.42	0	0	0	0	0	0	0	0
0.24	77.355	2.526	0	0	0	0	0	0	0	0
0.27	81.452	2.2105	0	0	0	0	0	0	0	0
0.3	75.952	3.2185	0	0	0	0	0	0	0	0
0.135	23.597	13.895	12.58	10.85	5.8341	5.1635	3.996	2.966	1.45	2.129
0.12	19.347	0.3738	0.409	0.684	0.8434	0.5387	0.446	0.551	0.689	0.773
0.105	1.0225	0.1368	0.144	0	0.4131	0.2799	0	0.135	0.146	0.285
0.09	0	0.1839	0.191	0	0.1771	0	0	0.185	0	0
0.075	0	0	0	0	0	0	0.23	0	0	0

Table S8. Population stability for different values of the parameter 'minimum cutoff resource for fertility.' The first row of each table corresponds to the baseline parameters, and the other rows correspond to trials with the parameter P systematically varying across its feasible range. Results are shown for egalitarian (E) and all nine stratified (H) population types.

P	E	H2	H3	H4	H5	H6	H7	H8	H9	H10
24.5	53.839	8.67	6.751	5.792	4.9765	2.8567	2.199	1.79	1.053	0.606
25	55.777	6.1095	7.088	4.925	5.377	3.428	2.744	2.032	0.724	0.925
25.5	84.7	7.2185	6.964	5.081	4.45	2.9085	1.861	1.938	1.273	0.842
26	84.763	7.0615	6.148	5.575	4.408	3.4615	2.453	1.202	0.76	0.831
26.5	84.501	6.5725	8.214	5.667	5.111	3.315	2.041	1.873	0.93	0.652
27	85.01	8.229	7.366	6.102	4.3612	3.876	2.402	1.823	1.547	1.128
24	84.752	8.6495	6.411	5.593	5.0675	2.264	2.091	1.915	1.149	0.761
23.5	84.211	7.946	7.312	5.305	4.4183	2.8055	2.39	1.901	0.929	0.715
23	84.162	8.193	6.803	4.918	6.4635	2.68	2.972	1.697	0.947	0.802
22.5	84.53	7.908	7.287	7.558	4.027	3.37	2.353	1.919	1.273	0.887
22	84.338	6.596	6.669	5.23	3.7145	3.7319	2.316	1.568	1.373	1.056

Table S9. Population stability for different values of the parameter 'maximum possible survival probability based on resources.' The first row of each table corresponds to the baseline parameters, and the other rows correspond to trials with the parameter P systematically varying across its feasible range. Results are shown for egalitarian (E) and all nine stratified (H) population types.

P	E	H2	H3	H4	H5	H6	H7	H8	H9	H10
0.998	53.839	8.67	6.751	5.792	4.9765	2.8567	2.199	1.79	1.053	0.606
0.999	87.38	2.935	3.104	2.577	2.7095	2.238	1.66	1.427	1.037	0.604
1	89.454	1.2695	0.821	1.034	1.267	1.215	1.2	0.581	1.033	0.43
0.994	12.996	0.5117	0.818	0.226	0.7396	0.2278	0.171	0.552	0.551	0.239
0.99	0	0	0	0	0	0	0	0	0	0
0.985	0	0	0	0	0	0	0	0	0	0
0.98	0	0	0	0	0	0	0	0	0	0
0.975	0	0	0	0	0	0	0	0	0	0
0.97	0	0	0	0	0	0	0	0	0	0
0.965	0	0	0	0	0	0	0	0	0	0
0.96	0	0	0	0	0	0	0	0	0	0

Table S10. Population stability for different values of the parameter ‘cutoff resources for survival.’ The first row of each table corresponds to the baseline parameters, and the other rows correspond to trials with the parameter P systematically varying across its feasible range. Results are shown for egalitarian (E) and all nine stratified (H) population types.

P	E	H2	H3	H4	H5	H6	H7	H8	H9	H10
22.5	53.839	8.67	6.751	5.792	4.9765	2.8567	2.199	1.79	1.053	0.606
23	84.634	8.144	7.343	6.108	4.9155	3.7351	2.414	1.934	1.192	0.718
23.5	83.972	9.7165	9.75	6.994	5.188	2.7495	2.793	2.1	1.829	0.639
24	84.649	9.9635	7.651	6.942	5.3001	4.0296	2.959	2.366	1.079	1.034
24.5	84.948	12.003	9.72	7.445	5.8038	3.3324	2.993	1.839	0.971	1.123
25	83.805	12.897	10.17	7.782	5.9737	4.6534	3.006	2.021	1.322	0.735
22	84.621	7.0218	7.089	4.888	5.235	3.6684	2.65	1.334	1.039	0.783
21.5	84.432	5.4705	5.707	3.767	3.6505	2.4182	2.324	1.57	0.941	0.951
21	84.78	5.879	4.782	5.098	4.5735	2.825	2.617	1.338	1.019	0.897
20.5	85.162	6.3325	6.917	4.683	2.8405	3.289	1.964	1.532	1.006	0.517
20	84.356	5.167	4.818	5.34	3.7745	3.601	1.967	1.346	0.9	1.007

Table S11. Population stability for different values of the parameter ‘resource productivity rate.’ The first row of each table corresponds to the baseline parameters, and the other rows correspond to trials with the parameter P systematically varying across its feasible range. Results are shown for egalitarian (E) and all nine stratified (H) population types.

P	E	H2	H3	H4	H5	H6	H7	H8	H9	H10
5	53.839	8.67	6.751	5.792	4.9765	2.8567	2.199	1.79	1.053	0.606
4.5	85.467	7.8623	6.876	4.344	3.4676	2.4129	1.501	1.327	0.959	0.417
4	86.05	7.712	6.433	4.316	2.5077	1.4895	0.619	0.622	0.672	0.253
3.5	86.234	7.71	4.856	3.358	2.0618	1.085	0.766	0.272	0.114	0.174
3	84.52	4.6225	3.203	1.603	1.1659	0.3232	0.226	0.114	0	0
2.5	79.196	3.5742	1.859	0.885	0.347	0.1174	0.171	0.116	0.067	0
6	82.781	6.5685	7.107	6.92	6.0725	4.108	3.525	2.896	2.504	1.825
7	80.907	5.9665	7.523	6.596	6.4025	6.494	4.717	3.508	3.662	2.142
8	79.348	5.86	5.867	7.105	6.818	5.6205	6.428	4.538	4.505	3.582
9	78.058	4.8459	6.22	7.331	7.468	7.556	7.402	5.79	4.905	4.522
10	77.05	3.884	5.947	6.03	7.037	7.8316	6.586	6.648	6.05	5.555

Table S12. Migration results for trials in which the same trigger was used for both society types. Unshaded columns show the total number of egalitarian (E) and stratified (H) societies generated during the 10,000-year trial, the number of each type which went extinct, the total numbers of each society type left at the end; the ratio of number of migrations for E vs H; the percent of populations going extinct for E and for H; and the first year in which all 100 sites were occupied. Shaded columns show the values used for the three trigger types (% Decline in 2 Years, Resource Deprivation, and Total Resource Depletion), and trial code numbers for constant and variable environment trials.

COMPETITION TRIALS FOR THREE TRIGGER TYPES, THREE VALUES -- SAME FOR EACH												
E # socs	H # socs	E extincts	H extincts	E at end	H at end	% DECLINE in 2 yrs	E:H migration ratio	% extinction E	% extinction H	1st yr all 100	TRIAL	
103	1134	4	1133	99	1	0.08	0.09	0.04	1.00	766	402	constant
56	166	0	122	56	44	0.13	0.34	0	0.73	1402	403	constant
61	102	0	63	61	39	0.18	0.60	0	0.62	1366	404	constant
191	4762	178	4675	13	87	0.08	0.04	0.93	0.98	939	412	variable
31	813	31	713	0	100	0.13	0.04	1.00	0.88	1412	413	variable
64	417	64	317	0	100	0.18	0.15	1.00	0.76	1836	414	variable
E # socs	H # socs	E extincts	H extincts	E at end	H at end	RES DEPRIVATION	E:H migration ratio	% extinction E	% extinction H	1st yr all 100	TRIAL	
51	124	0	75	51	49	36	0.41	0.00	0.60	1408	502	constant
54	132	1	85	53	47	34	0.41	0.02	0.64	1521	503	constant
54	116	0	70	54	46	32	0.47	0.00	0.60	1493	504	constant
57	385	57	285	0	100	36	0.15	1.00	0.74	1769	512	variable
57	437	57	337	0	100	34	0.13	1.00	0.77	1618	513	variable
59	437	59	337	0	100	32	0.14	1.00	0.77	1716	514	variable
E # socs	H # socs	E extincts	H extincts	E at end	H at end	Total Res Depletion	E:H migration ratio	% extinction E	% extinction H	1st yr all 100	TRIAL	
53	47	0	0	53	47	45000	1.13	0.00	0.00	668	601	constant
36	68	0	4	36	64	30000	0.53	0.00	0.06	773	602	constant
81	49	2	28	79	21	15000	1.65	0.02	0.57	1388	603	constant
48	114	48	14	0	100	45000	0.42	1.00	0.12	721	611	variable
32	163	32	63	0	100	30000	0.20	1.00	0.39	846	612	variable
39	236	39	136	0	100	15000	0.17	1.00	0.58	2124	613	variable

Table S13. Migration results for trials in which the optimal trigger was used for each population type. Unshaded columns show the total number of egalitarian (E) and stratified (H) populations generated during the 10,000-year trial, the number of each type which went extinct, the total numbers of each population type left at the end; the ratio of number of migrations for E vs H; the percent of populations going extinct for E and for H; and the first year in which all 100 sites were occupied. Shaded columns show the values used for the three trigger types (% Decline in 2 Years, Resource Deprivation, and Total Resource Depletion) for E and H, and trial code numbers for constant and variable environment trials.

OPTIMAL TRIGGER VALUE COMPETITION TRIALS FOR THREE TRIGGER TYPES

E # socs	H # socs	E extincts	H extincts	E at end	H at end	% DECLINE in 2 yrs	E:H migration ratio	% extinction E	% extinction H	1st yr all 100	TRIAL	
67	246	4	209	63	37	E .08, H .13	0.27	0.06	0.85	1181	721	CONSTANT
29	804	29	704	0	100	E .08, H .13	0.04	1.00	0.88	1643	731	VARIABLE

E # socs	H # socs	E extincts	H extincts	E at end	H at end	RES DEPRIVATION	E:H migration ratio	% extinction E	% extinction H	1st yr all 100	TRIAL	
52	148	0	100	52	48	E 32, H 36	0.35	0.00	0.68	1566	821	CONSTANT
95	378	95	278	0	100	E 36, H 34	0.25	1.00	0.74	1537	831	VARIABLE

E # socs	H # socs	E extincts	H extincts	E at end	H at end	Total Res Depletion	E:H migration ratio	% extinction E	% extinction H	1st yr all 100	TRIAL	
19	86	0	5	19	81	E 15000, H 30000	0.22	0.00	0.06	848	901	CONSTANT
19	170	19	70	0	100	E 15000, H 30000	0.11	1.00	0.41	1005	911	VARIABLE

Table S14. Evaluating Support for Predictions Using Case Studies

	1 unequal	2 unstable	2.5 stratified	3 migration	3.5 stratified	4 conflict	4.5 stratified	5 variable	6 surplus	7 expansion
a) Levant	2	1	2	0	0	0	0	0	3	3
b) Mesopotamia	1	1	2	2	0	2	2	0	0	0
c) Oaxaca	1	1	1	1	1	1	1	0	2	1
d) N. Channel Is.	0	1	2	1	0	1	0	0	0	0
e) S. Cal. Bight	2	1	2	0	0	1	1	2	0	0
f) Kodiak	1	1	1	0	0	1	1	0	1	0
g) NW Coast	2	2	2	0	0	1	1	0	1	0
h) Intermontane	1	1	1	0	0	1	1	3	1	0
i) Mesa Verde	0	1	0	1	0	1	0	0	0	1
j) Madagascar	2	2	0	1	0	1	1	0	0	2
k) Tonga	1	2	0	1	2	0	0	0	0	2
l) Hawaii	1	1	0	1	0	1	1	0	1	1
m) Easter Is.	1	1	0	1	1	1	1	0	0	1
n) New Zealand	0	2	0	1	0	1	0	0	0	2
o) Polynesia	1	1	0	1	1	1	1	1	1	1

Evaluation Criteria

1. Do stratified societies exhibit unequal allocation of resources (in addition to status differences)?
2. Do populations grow, overshoot carrying capacity, and then decline?
2.5 Is this phenomenon more associated with stratified societies?
3. Do growing populations expand through migration to additional sites?
3.5 Is this phenomenon more associated with stratified societies?
4. Is conflict associated with expansion to new sites, once the landscape is populated?
4.5 Is this phenomenon more associated with stratified societies?
5. Are stratified societies associated with more variable environments?
6. Are stratified societies associated with more storage of surplus?
7. Did stratified societies increase in frequency through expansion and conquest (as opposed to internal change)?

Metrics

0 no clear indication for or against
1 definitely observed
2 partially observed
3 partially contradicted
4 definitely contradicted

Case Studies

a) South-Central Levant: Kuijt 2000; Kuijt 2008
b) Mesopotamia: Wright & Johnson 1975; Wright et al. 1975
c) Oaxaca (Central America): Wright 1977; Flannery & Marcus 2000; Flannery & Marcus 2003; Spencer 2003
d) Northern Channel Islands (North America): Kennett et al. 2009
e) Southern California Bight (North America): Ames 2004
f) Kodiak Archipelago (North America): Ames 2004
g) Northwest Coast (North America): Ames 2004
h) Intermontane Plateau (North America): Ames 2004
i) Mesa Verde Region (North America): Kohler et al. 2009
j) Madagascar: Dewar & Wright 1993
k) Tonga (Polynesia): Kirch 1984
l) Hawaii (Polynesia): Kirch 1984
m) Easter Island (Polynesia): Kirch 1984
n) New Zealand (Polynesia): Walter et al. 2006
o) Polynesia: Kirch 1984; Younger 2008; Kirch & Rallu 2007; Kennett & Winterhalder 2008
